# Gene Regulatory Mechanisms Underlying the Spatial and Temporal Regulation of Target-Dependent Gene Expression in *Drosophila* Neurons

**DOI:** 10.1371/journal.pgen.1005754

**Published:** 2015-12-29

**Authors:** Anthony J. E. Berndt, Jonathan C. Y. Tang, Marc S. Ridyard, Tianshun Lian, Kathleen Keatings, Douglas W. Allan

**Affiliations:** 1 Department of Cellular and Physiological Sciences, University of British Columbia, Vancouver, British Columbia, Canada; 2 Department of Genetics, Harvard Medical School, Boston, Massachusetts, United States America; Washington Universtiy, UNITED STATES

## Abstract

Neuronal differentiation often requires target-derived signals from the cells they innervate. These signals typically activate neural subtype-specific genes, but the gene regulatory mechanisms remain largely unknown. Highly restricted expression of the FMRFa neuropeptide in *Drosophila* Tv4 neurons requires target-derived BMP signaling and a transcription factor code that includes Apterous. Using integrase transgenesis of enhancer reporters, we functionally dissected the Tv4-enhancer of *FMRFa* within its native cellular context. We identified two essential but discrete *cis*-elements, a BMP-response element (BMP-RE) that binds BMP-activated pMad, and a homeodomain-response element (HD-RE) that binds Apterous. These *cis*-elements have low activity and must be combined for Tv4-enhancer activity. Such combinatorial activity is often a mechanism for restricting expression to the intersection of *cis*-element spatiotemporal activities. However, concatemers of the HD-RE and BMP-RE *cis*-elements were found to independently generate the same spatiotemporal expression as the Tv4-enhancer. Thus, the Tv4-enhancer atypically combines two low-activity *cis*-elements that confer the same output from distinct inputs. The activation of target-dependent genes is assumed to 'wait' for target contact. We tested this directly, and unexpectedly found that premature BMP activity could not induce early *FMRFa* expression; also, we show that the BMP-insensitive HD-RE *cis*-element is activated at the time of target contact. This led us to uncover a role for the nuclear receptor, *seven up (svp)*, as a repressor of *FMRFa* induction prior to target contact. Svp is normally downregulated immediately prior to target contact, and we found that maintaining Svp expression prevents *cis*-element activation, whereas reducing *svp* gene dosage prematurely activates *cis*-element activity. We conclude that the target-dependent *FMRFa* gene is repressed prior to target contact, and that target-derived BMP signaling directly activates *FMRFa* gene expression through an atypical gene regulatory mechanism.

## Author Summary

Nerve cells extend long processes that grow out to contact the target cells with which they communicate. When the nerve cell makes initial contact, the target cells send a retrograde signal back to the nerve cell. Such target-derived signals activate and maintain important genes that make the nerve cell functional, such as genes determining neurotransmitter type. This is a well-characterized phenomenon throughout the nervous systems of flies to mammals, but we still do not know how these signals actually activate gene expression. We now provide details regarding target-dependent signal regulation of nerve cell genes. We model this in Tv4 neurons of *Drosophila melanogaster*, which require target-derived BMP signaling to trigger FMRFa neuropeptide expression. Our study shows how DNA-binding transcription factors of the BMP signaling pathway integrate with other transcription factors at specific regulatory DNA sequences to activate *FMRFa* expression, and define the atypical logic by which this occurs. We also provide novel insight into how target-dependent genes are regulated before target contact. Instead of simply waiting for target-dependent activation, these genes seem to be blocked from being expressed prior to target contact. These findings have relevance to mammals because the role of target-derived BMP signaling in nerve cell gene regulation is conserved between vertebrates and invertebrates.

## Introduction

Nervous system development requires the differentiation of diverse neuronal subtypes under the direction of combinatorially acting transcription factors [1, 2]. However, target-derived signaling from axo-dendritic targets, in the form of retrograde bone morphogenetic protein (BMP), transforming growth factor β (TGFβ), neurotrophin, or cytokine signaling, is often required to terminally differentiate a neuron's identity, mature morphology or function [3–6]. Target-dependent genes are often neurotransmitter enzymes or neuropeptides that mediate intercellular communication [7–13], or ion channels that mediate mature physiological properties [14, 15]. In addition, target-derived signaling can induce subtype-specific transcription factor profiles that drive branching of axo-dendritic arbors or appropriate topographic mapping of projections [16–19].

Strong genetic and cellular data supports a role for target-derived signaling in triggering target-dependent and neuronal subtype-specific gene transcription, yet our current view is not well informed by an understanding of the underlying gene regulatory mechanisms. Two broad possibilities have been discussed regarding the role of pleiotropic target-derived signals in triggering subtype-specific gene expression [3, 4]. First, they may contribute by promoting the activity of established transcriptional complexes that pre-determine gene expression. Alternatively, dedicated signaling pathway transcription factors might bind *cis*-regulatory sequences and contribute alongside cell-specific transcription factors to combinatorially specify gene expression. Here, we examined the gene regulatory mechanisms of target-derived signaling by examining how target-derived BMP signaling triggers *FMRFa* gene expression selectively in *Drosophila* Tv4 neurons.

In *Drosophila*, target-derived BMP signaling positively regulates neuromuscular synaptic morphology, transmission and plasticity [20–23], as well as subtype-specific neuropeptide gene expression [12, 13, 24]. *Drosophila* neuronal BMP signaling is induced by the postsynaptic-secreted Glass Bottom Boat (Gbb) ligand that acts at presynaptic BMP receptors Wishful thinking (Wit), Thickveins (Tkv) and Saxophone (Sax) [13, 20–22]. The type I BMP-receptors, Tkv and Sax, phosphorylate the receptor Smad, Mad (pMad; vertebrate Smad 1/5/8), which then couples with its co-Smad, Medea (vertebrate Smad 4) that together can act as sequence-specific transcription factors, or as transcriptional co-regulators [25–28]. The activities of the BMP and the closely-related TGFβ pathways can diverge from all levels of this linear pathway and feed into other signal transduction or miRNA pathways, providing multiple avenues by which BMP signaling could influence gene regulation [29–32].

**Fig 1:**
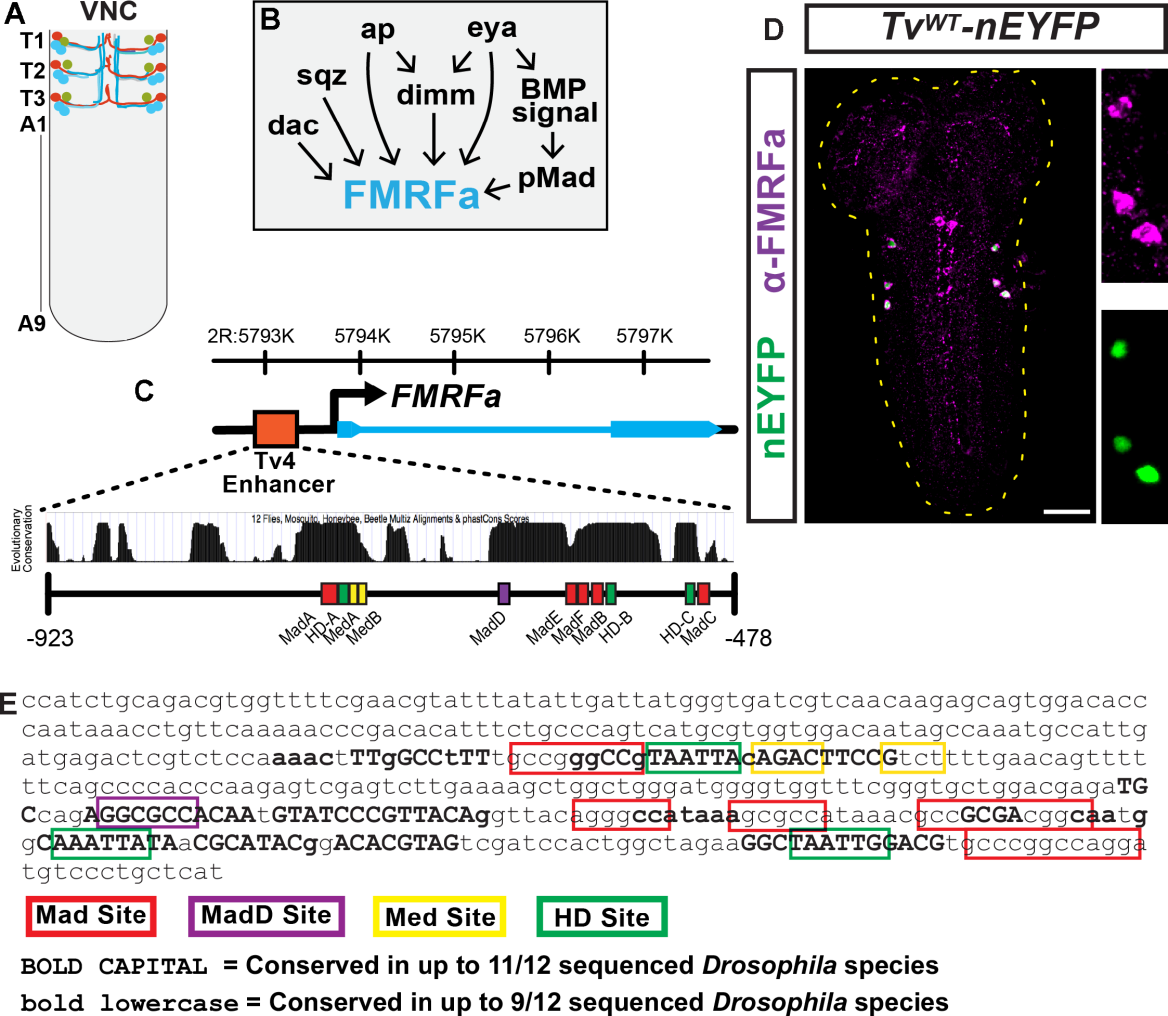
The Tv4-enhancer faithfully reports *FMRFa* expression exclusively in Tv4 neurons and contains conserved putative binding sites for Ap and Smads. (**A**) Tv neurons in the embryonic/larval VNC; Tv1 neurons (green), Tv2/3 neurons (blue) and Tv4 neurons (red). Tv1 neurons express the neuropeptide Nplp1 and Tv4 neurons express FMRFa. Segment number indicated on the left side of the VNC. (**B**) Transcription factors postulated to regulate *FMRFa* in Tv4 neurons. (**C**) Genome coordinates (Release 5) and scale image of *FMRFa* gene locus (exons denoted by thick blue lines, introns denoted by thin blue line, promoter denoted by arrow) and the 445 bp Tv4-enhancer (red box). Below is a conservation histogram through the Tv4-enhancer across 12 *Drosophila* species (high peaks = best conserved) from UCSC Browser. Below that, we show the relative location of putative homeodomain (green box), Mad (red and magenta boxes) and Medea sites (yellow boxes). **(D**) Nuclear-localized EYFP reporter expression driven from the wildtype 445 bp Tv4-enhancer (*Tv* ^*WT*^ *-nEYFP*). *Tv* ^*WT*^ *-nEYFP* is only expressed in Tv4 neurons (side panels; anti-FMRFa upper panel, *Tv* ^*WT*^ *-nEYFP* lower panel). Scale bar is 30 μm. (**E**) Sequence of *Drosophila melanogaster* Tv4-enhancer showing putative Homeodomain (green box), Mad (red or magenta box) and Medea (yellow box) binding sites. Conservation of nucleotide identity was identified using the Relaxed EvoPrint (EvoprinterHD), and is shown here using two layers of conservation. The first layer is shown by bolded capital letters to denote nucleotides conserved in 11 of 12 sequenced *Drosophila* species (see **S4 Fig**). The second layer is shown as small bolded letters that are conserved in 9 of 12 *Drosophila* species.

The *Drosophila* ventral nerve cord (VNC) has one Tv4 neuron in each of the six thoracic hemisegments. These six Tv4 neurons express the neuropeptide gene *FMRFa* that encodes a prepropeptide (FMRFa). The FMRFa prepropeptide is processed to multiple amidated FMRFamide neuropeptides (FMRFamide), which facilitate neurotransmission at the neuromuscular junction, a mechanism required for behaviours such as escape responses [33–36]. Tv4 neurons are born at embryonic stage (Stg.) 14, and their axons innervate the ipsisegmental dorsal neurohaemal organ (DNH) in mid to late Stg. 17 embryos (**Fig 1A**). Tv4 axons gain access to Gbb at their target. Gbb activates a retrograde BMP signaling that is absolutely essential for *FMRFa* gene initiation and maintenance throughout the organism's life [13, 37]. A logical genetic explanation for the extreme specificity of *FMRFa* expression is provided by genetic analysis showing that *FMRFa* expression requires BMP signaling and a Tv4-specific combination of transcription factors (TFs); the sequence-specific TFs Apterous (Ap), Squeeze (Sqz), Dimmed (Dimm) and Grainy head (Grh), and the transcriptional co-regulators Eyes absent (Eya) and Dachshund (Dac). In gain-of-function studies, a combination of Ap, Dac and BMP-signaling is sufficient to induce strong ectopic *FMRFa* gene expression in other neurons [13, 38–42] (**Fig 1B**).

We now address how BMP-signaling acts in relation to these known transcriptional regulators to initiate *FMRFa* gene expression. We identified necessary *cis*-elements within a 445 bp Tv4-specific *FMRFa* enhancer (including the homeodomain response element, HD-RE and the BMP-response element, BMP-RE), characterized transcriptional inputs that act at these two *cis*-elements, and provide an understanding of the developmental information that these two *cis*-elements contribute to shape *FMRFa* spatiotemporal expression [42, 43]. We show that induction of the *FMRFa* gene requires activation of the discrete HD-RE and BMP-RE *cis*-elements. Ap binds and *trans*-activates from the HD-RE, while BMP-activated Smads bind and *trans*-activates from the BMP-RE. Ap coordinates both *cis*-elements by virtue of its additional indirect regulation of the BMP-RE. Both *cis*-elements independently generate proper spatial expression, but because both *cis*-elements have low activity they must be simultaneously activated to generate Tv4-enhancer activity. Finally, we find that proper temporal initiation of *FMRFa* is produced by an unanticipated bipartite mechanism. Prior to target contact, the nuclear receptor Svp represses both *cis*-elements. Svp is downregulated immediately prior to target contact, which de-represses the HD-RE and permits the subsequent BMP-dependent activation of the BMP-RE upon target contact. The coordinate de-repression and activation of the HD-RE and BMP-RE in the late embryo then leads to Tv4-enhancer activation and *FMRFa* expression.

## Results

### The Tv4-enhancer responds appropriately to *FMRFa* transcriptional regulators

A 445 bp *cis-*regulatory region upstream from the *FMRFa* gene, that we term the Tv4-enhancer, is sufficient to drive reporter expression exclusively in Tv4 neurons [42, 43]. Tv4-enhancer reporter activity requires *apterous*, and three candidate Apterous binding sites were postulated to mediate this function [42, 43]. We PCR-amplified the Tv4-enhancer from Oregon R and placed it into a phiC31-integrase-compatible transgenic nEYFP reporter vector, to generate a *Tv* ^*WT*^ *-nEYFP* reporter transgene integrated into *attP2* (**Fig 1C, 1D and 1E**). We found that *Tv* ^*WT*^ *-nEYFP* expression faithfully reported FMRFa neuropeptide expression in Tv4 neurons (Fig 1D).

**Fig 2:**
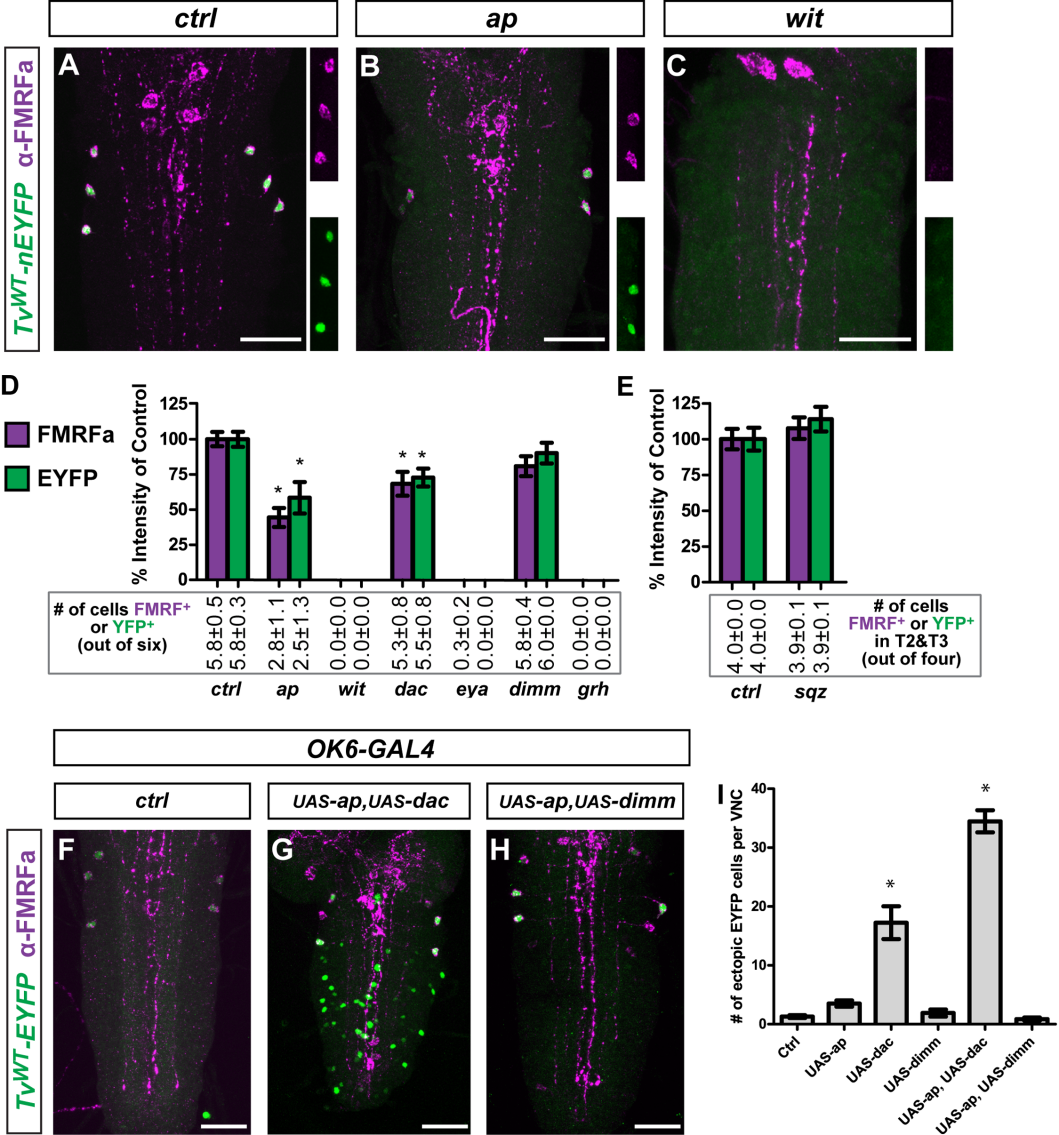
The Tv4-enhancer responds appropriately to known *FMRFa* transcriptional regulators. (**A-C**) Representative images of *Tv* ^*WT*^ *-nEYFP* (green) and FMRFa immunoreactivity (magenta) in controls (*ctrl*), and in *ap* and *wit* mutants (side panels; FMRFa upper panel, *Tv* ^*WT*^ *-nEYFP* lower panel), showing partial and full loss of reporter and FMRFa peptide expression, respectively. **(D**) Graph showing % fluorescence intensity for FMRFa immunoreactivity and *Tv* ^*WT*^ *-nEYFP* reporter expression in mutant backgrounds for known regulators of *FMRFa*, relative to the mean of the control. Numbers below columns represent the number of Tv4 neurons that express detectable FMRFa or *Tv* ^*WT*^ *-nEYFP*. (**E**) In *sqz* mutants, T1 segment Tv4 neurons are often not generated, so we show intensity and FMRFa expression for T2 and T3 segments only. (**D,E**) All data represented as mean±SEM. Data compared using one-way ANOVA with Tukey HSD *post-hoc* test. * = p<0.05 compared to controls. n = 10–20 animals per genotype. (**F-H**) *OK6-GAL4* drove combinations of *UAS-ap* with either *UAS-dac* or *UAS-dimm*. Representative images of *Tv* ^*WT*^ *-nEYFP* and FMRFa expression in whole VNCs are shown; these were imaged through the entirety of their z-axis. **(I)** The number of neurons expressing ectopic nEYFP was counted when we overexpressed *ap*, *dac* or *dimm* in the combinations shown from *OK6-GAL4*. Only *UAS-dac* or *UAS-ap*,*UAS-dac* together induced ectopic *Tv* ^*WT*^ *-nEYFP*-positive cells. Data is represented as mean number of ectopic nEYFP cells ± SEM. n = 10–20 VNCs per genotype. Data compared using one-way ANOVA with Tukey HSD *post-hoc* test. * = p<0.001 compared to controls. Scale bars are 30 μm in all images. **Genotypes in A-E: *ctrl (*** *Tv* ^*WT*^ *-nEYFP)*. ***wit*** *(Tv* ^*WT*^ *-nEYFP*,*wit* ^*A12*^ */ Tv* ^*WT*^ *-nEYFP*,*wit* ^*B11*^ *)*. ***ap***, *(ap* ^*GAL4*^ */ ap* ^*P44*^ *; Tv* ^*WT*^ *-nEYFP)*. ***sqz*** *(Tv* ^*WT*^ *-nEYFP*,*sqz* ^*ie*^ */ Tv* ^*WT*^ *-nEYFP*,*sqz* ^*ie*^ *)*. ***dac*** *(Df(2L)Exel7066/ dac* ^*3*^ *; Tv* ^*WT*^ *-nEYFP)*. ***eya*** *(eya* ^*Cli-IID*^ */eya* ^*D1*^ *; Tv* ^*WT*^ *-nEYFP)*. ***grh***, *(grh* ^*IM*^ */grh* ^*Df*^ *; Tv* ^*WT*^ *-nEYFP)*. ***dimm*** *(dimm* ^*rev4*^ */dimm* ^*P1*^ *; Tv* ^*WT*^ *-nEYFP)*.

We examined *Tv* ^*WT*^ *-nEYFP* activity in early larval stage 1 (L1) larvae that were mutant for regulators known to affect *FMRFa* gene expression. We quantified the number (per VNC) of Tv4 neurons expressing nEYFP, as well as the relative intensity of nEYFP in individual Tv4 neurons (normalized to the mean of the control) (**Fig 2A, 2B and 2C**). Loss of BMP signaling in *wishful thinking* (*wit*) type II BMP receptor nulls eliminated FMRFa immunoreactivity and *Tv* ^*WT*^ *-nEYFP* expression (**Fig 2C and 2D**). In strong *ap* hypomorphs, *Tv* ^*WT*^ *-nEYFP* was expressed in ~2.5 Tv4 neurons per VNC at 58% of control intensity; comparable to the reduction in FMRFa immunoreactivity (**Fig 2B and 2D**). The co-regulator *dac* is only modestly required for *FMRFa* expression in embryos, but its overexpression upregulates *FMRFa*, and it acts combinatorially with *apterous* to trigger ectopic *FMRFa* in BMP-activated motoneurons [41]. In correspondence, in *dac* nulls, *Tv* ^*WT*^ *-nEYFP* was expressed in ~5.5 Tv4 neurons per VNC at 72% of control intensity (**Fig 2D**). Overexpression of *UAS-dac* in Tv4 neurons (by *ap* ^*GAL4*^) upregulated *Tv* ^*WT*^ *-nEYFP* to 144±10% of control levels (p<0.01 two-tailed t-test, n = 48 and n = 56 Tv4 neurons for control and overexpression, respectively). Also, ectopic *Tv* ^*WT*^ *-nEYFP* expression was activated in motoneurons by *OK6-GAL4-*driven misexpression of *UAS-dac* alone, or *UAS-dac* and *UAS-ap* together (**Figs 2G, 2I** and S1). The co-regulator *eya* is essential for *FMRFa* expression [41], and *Tv* ^*WT*^ *-nEYFP* was entirely eliminated in strong *eya* hypomorphs in late Stg. 17 embryos (**Figs 2D and S1**). The temporal transcription factor, *grh*, is required for generation of Tv4 neurons but its expression is reduced by the time of *FMRFa* expression [38, 44]. Predictably, *Tv* ^*WT*^ *-nEYFP* expression was eliminated in *grh* nulls (**Figs 2D and S1**). Thus, BMP-signaling, *ap*, *dac* and *eya* are regulators of the Tv4-enhancer in postmitotic Tv4 neurons.

Previous evidence suggested that *FMRFa* is *sqz* and *dimm*-dependent [45]. Here, our data suggest that this regulation is not directly at the transcriptional level. In *sqz* nulls, we verified that Tv4 neurons are often not generated in the T1 segment, and that supernumerary Nplp1-expressing Tv1 neurons are generated in Tv clusters (**S2 Fig**) [38–40]. We quantified *Tv* ^*WT*^ *-nEYFP* expression in segments T2 and T3, but found no effect on FMRFa immunoreactivity or *Tv* ^*WT*^ *-nEYFP* in *sqz* mutants (**Fig 2E**). In *dimm* mutants, FMRFamide immunoreactivity was 47% of control levels (p<0.001 Two-tailed t-test, n = 48 and n = 36 Tv4 neurons for *dimm* ^*Rev4*^ */dimm* ^*P1*^ *and dimm* ^*Rev4*^ */+* controls, respectively). In contrast, there was no reduction in the FMRFa prepropeptide or in *Tv* ^*WT*^ *-nEYFP* (**Fig 2D**). Also, *Tv* ^*WT*^ *-nEYFP* was not ectopically activated when we co-misexpressed *UAS-ap* and *UAS-dimm* in all motoneurons, using *OK6-GAL4* (**Fig 2H and 2I**). This corresponds to our previous findings in adults showing that *dimm* knockdown eliminated FMRFamide but not FMRFa prepropeptide or *FMRFa* transcript [37]. Thus, we eliminate *grh*, *sqz* and *dimm* as direct regulators of the Tv4-enhancer.

### The Tv4-enhancer has conserved sequences matching homeodomain and Smad binding motifs

Our genetic analyses found that Ap, BMP signaling, Dac and Eya regulate Tv4-enhancer activity. As only Ap and BMP-activated Smads are sequence-specific transcription factors, we looked for potential binding motifs in the Tv4 enhancer. DNA sequence motifs for binding of Apterous and the Smads, Mad and Medea have been determined [42, 46–48]. Candidate sequences matching these motifs were identified within the Tv4-enhancer; and using phastCONS in the UCSC Genome Browser and EvoprinterHD [49] across 12 *Drosophila* species (**Figs 1C, 1E, S3 and S4**). These include three previously-described, putative Apterous motifs (HD-A,B,C) [42] (**Figs 1C, 1E, S3 and S4**), and six GC-rich sequences with some similarity to Mad motifs (Mad-A-F) [50, 51] (**Figs 1C, 1E, S3 and S4**). Of these, only Mad-D is perfectly conserved and precisely matches a characterized *Drosophila* Mad sequence [GGCGCCA] [47] (**Figs 1C,1E and S3**). *Drosophila* Mad and Medea typically act at a bipartite motif, such as [GGCGCCA(N_4_)GNCV] [47] or [GRCGNC(N_5_)GTCT] [48]. Only one region approximates either of these motifs; Mad-A is 6 bp from a GTCT sequence (Med-A), but this is inverted with respect to its typical orientation, and is 15 bp from a poorly conserved GTCT sequence, (Med-B) [**GGGCCG** TAATTAC**AGAC**TTCC**GTCT** **]** (**Figs 1E, S3 and S4**). The juxtaposition of the **Mad**, Ap, and **Med** bindings sites in this sequence was suggestive of an Ap/Smad integration site. Homeodomain TFs can act cooperatively or collaboratively with Smads at coupled motifs [52–56]. Such a model could account for restriction of *FMRFa* expression in Tv4 neurons, as Ap and BMP signaling in the VNC only coincide in Tv4 neurons.

### Two highly conserved *cis*-elements are necessary for Tv4-enhancer activity

**Fig 3:**
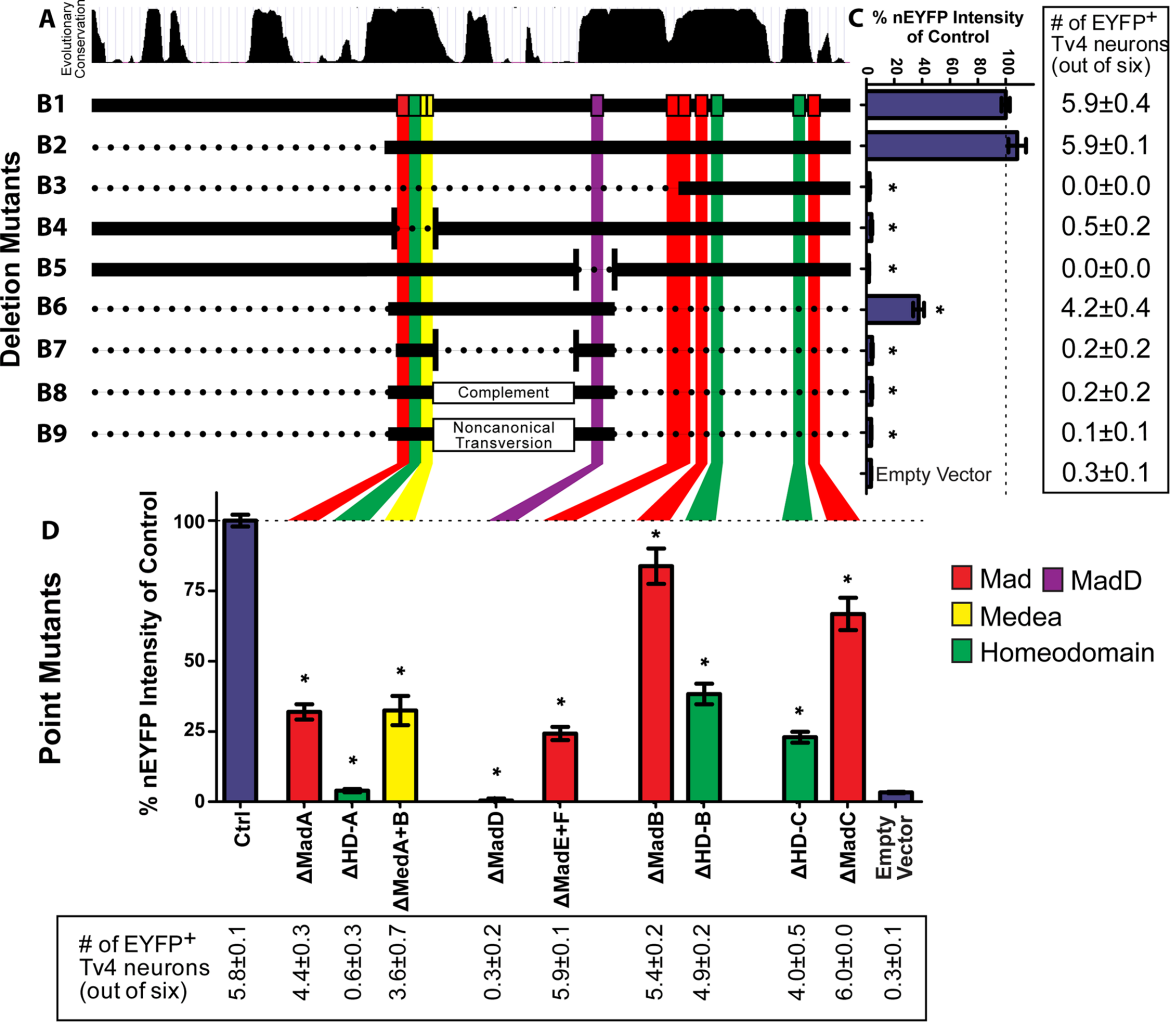
Mutant sequence analysis of the Tv4-enhancer. (**A**) Conservation track from UCSC Browser showing conservation islands between 12 sequenced *Drosophila* species. (**B1-B9**) Graphic representation of the position of putative HD (green), Mad (red and magenta) and Med motifs (yellow) as the coloured vertical bars within the Tv4-enhancer. These overlay the depicted deletion series of the Tv4-enhancer B1-B9. We generated a series of deletion transgenes (B1-B9; dotted region deleted). **B1** is the full length wild-type Tv4-enhancer, denoted by the complete horizontal solid black line. **B2-B9** show the region present in the reporter transgene (horizontal black bar) after removal of certain sequences (dotted regions). **B4,5,7** show a deletion between two regions; the points of fusion are shown by vertical bars, between which the intervening sequence is removed. **B8,9** show the type of sequence conversion performed within the region shown. (**C**) Reporter expression driven from control or deletion mutants B1-B9, or the empty reporter vector, expressed in the bar graph as the % nEYFP fluorescence intensity relative to mean of the B1 control. The right-most panel provides the number of Tv4 neurons (out of six) expressing significant nEYFP above the 99% confidence interval of the empty vector control. Removal of the HD-A motif (green, B3,B4) or the Mad-D motif (magenta, B3,B5) severely reduced expression. The enhancer fragment spanning the HD-A to Mad-D motifs (B6) expressed at moderate levels, but alteration of the intervening sequence severely reduced reporter expression, whether by sequence elimination (B7), conversion to complementary sequence (B8) or non-canonical sequence conversion (B9). (**D**) Point substitution mutants of Mad, HD and Med motifs, showing (bar graph) expression levels as % nEYFP fluorescence intensity relative to mean of the control, or (bottom-most panel) the number of Tv4 neurons (out of six) expressing significant nEYFP above the 99% confidence interval of the empty vector control. Only substitution mutants that alter the HD-A or Mad-D motifs essentially eliminate reporter expression. n = 10–20 animals per genotype. All data represented as mean±SEM. Data compared using one-way ANOVA with Tukey HSD *post-hoc* test. * = p<0.05 compared to controls.

To identify essential sequences in the Tv4-enhancer and to also directly test putative Ap and Smad binding motifs, we performed deletion and substitution studies of the Tv4-enhancer. We placed each mutant Tv4-enhancer reporter transgene into the genomic *attP2* site, to allow for quantitative comparison of all wildtype and mutant Tv4-enhancers, *in vivo* in their appropriate cellular context, the Tv4 neurons. Exact details of deletions and substitution mutation can be found in S1 Table. We quantified the number of Tv4 neurons that express nEYFP (in early L1 larvae), as well as fluorescence intensity normalized to the mean of the control (**Fig 3A, 3B and 3C**). A reporter-only empty vector control was used as the relative zero; Tv4 neurons with nEYFP reporter intensity above the upper 99% confidence interval for the empty vector control (9.7% of *Tv* ^*WT*^ *-nEYFP*) were counted as expressing nEYFP.

Reporter expression was reduced in most sequence deletions and mutants, but our combined analysis pinpointed two specific regions that are absolutely required for expression, and also contain putative Ap or Mad sequence motifs. Deletions that removed the short conservation islands containing either the HD-A or the Mad-D motifs eliminated expression (**Fig 3B3, 3B4 and 3B5**). Further, substitution mutants at the HD-A or Mad-D motif also eliminated reporter expression (**Fig 3D**). Thus, both HD-A and Mad-D *cis*-elements are required non-redundantly (**Fig 3B4 and 3B5**). Deletion of most regions outside these two *cis*-elements had partial or no effect on reporter expression. We also found that deletion of the low conservation region between HD-A and Mad-D abrogated reporter expression (**Fig 3B7**). To discriminate whether this region has informational content or acts as a simple spacer between HD-A and Mad-D, we mutated it in two ways; a complement sequence to maintain local GC/AT content, and also a non-canonical nucleotide transversion [57]. In both cases, reporter expression was abrogated (**Fig 3B8 and 3B9**). Thus, this intervening sequence is essential for expression and does not merely act to space HD-A and Mad-D to an appropriate distance. We conclude that the region spanning HD-A to Mad-D is absolutely critical for Tv4-enhancer activity. This region contains the critical HD-A homeodomain motif and the critical Mad-D Mad motif, that together flank a critical low conservation sequence with no predicted binding motif for known *FMRFa* regulators.

In the nervous system, the coincidental expression of Ap with BMP activity appears to be unique to Tv4 neurons. Also, overlap of Dac and BMP-signaling in ventral nerve cord neurons is extremely rare [12]. Functionally, misexpression of Ap, Dac in BMP-activated motoneurons, or co-misexpression of Ap, Dac and BMP activation, is sufficient for widespread ectopic FMRFa expression in neurons [41]. Thus, given that Ap, Dac and BMP are combinatorially necessary and sufficient for *FMRFa* expression, we hereafter focused on the two *cis*-elements containing the HD-A and Mad-D motifs, as these are likely critical integration sites through which Ap and BMP-signaling generate the Tv4-enhancer’s spatiotemporal expression.

### The HD-A motif is part of a homeodomain response element (HD-RE) that binds Ap, and the Mad-D motif is part of a BMP response element (BMP-RE) that binds Mad

**Fig 4:**
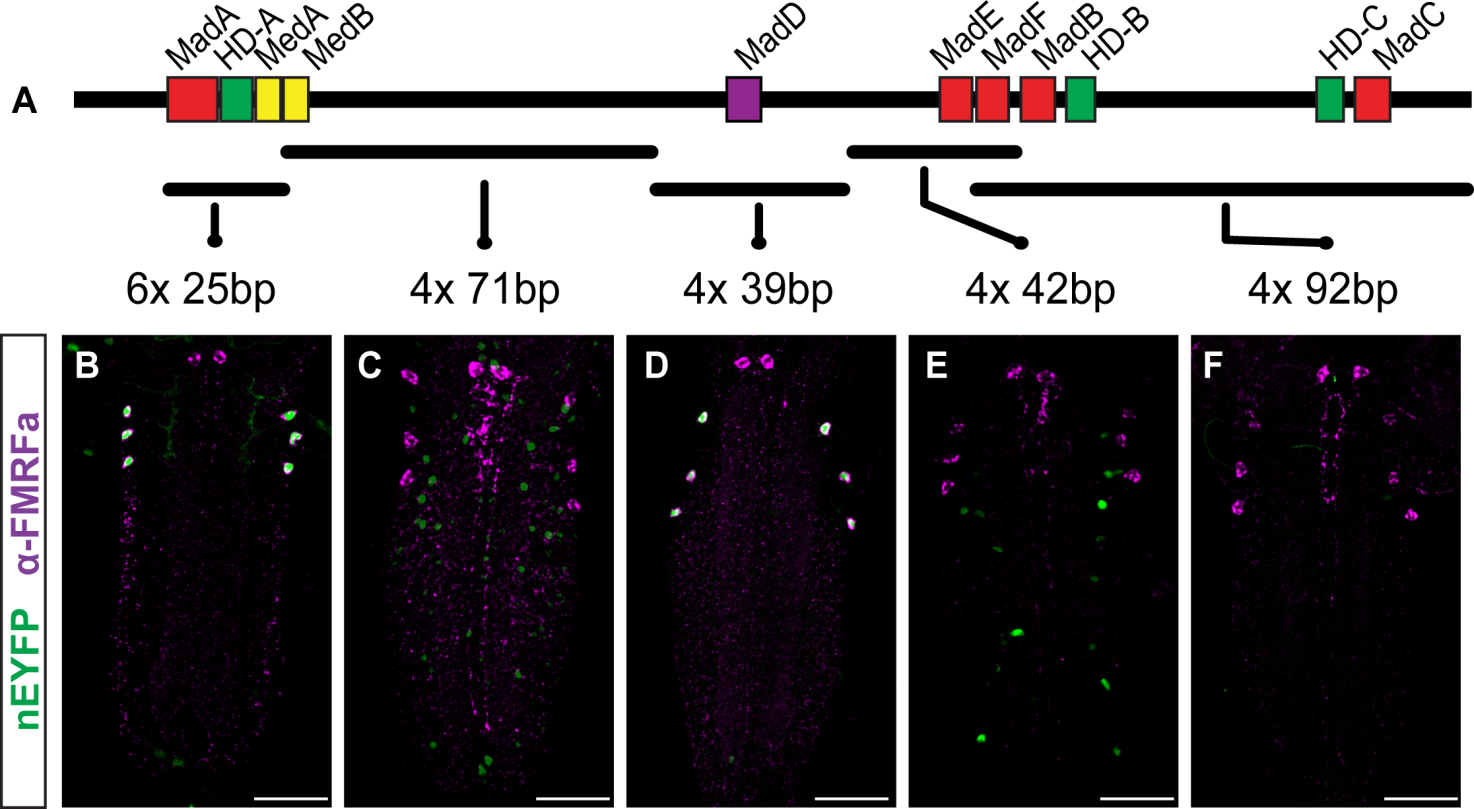
The 6xHD-A and 4xMad-D *cis*-elements encode sufficient information for Tv4-specific expression. (**A**) Relative position of putative HD, Mad and Medea motifs. We concatemerized fragments from the Tv4-enhancer (shown in A). The number of concatemeric direct sequence repeats is shown, as is the number of nucleotides within each direct repeat. (**B-F**) Reporter expression for each concatemer, shown above, in the early L1 VNC. Two regions generate reporter expression in Tv4 neurons, the 25 bp HD-A-containing conserved region (**B**) and the 39 bp Mad-D containing conserved region (**D**). All other regions generate weak widespread neuronal expression (**C**), ectopic expression (**E**), or fail to express (**F**). Scale bars are 30 μm in all images.

What *cis*-regulatory information is encoded by the HD-A- and Mad-D-containing *cis*-elements? The HD-A sequence is flanked by Mad-A and Med-A, representing a prime candidate site for cooperativity of Ap and BMP-activated Smads. To explore mechanisms for Ap and Smad integration, we tested reporter activity generated by the *cis*-elements containing HD-A and Mad-D. We placed the conserved island containing HD-A (25 bp spanning Mad-A, HD-A, Med-A) and Mad-D (39 bp), separately, into integrase-compatible reporter vectors to generate attP2-integrated transgenic reporter flies. Monomers of either *cis*-element failed to generate reporter activity. However, concatemeric repeats of either *cis*-element generated reporter activity in Tv4 neurons, with robust expression occurring in *6xHD-A-nEYFP* and *4xMad-D-nEYFP* reporters. Remarkably, expression generated from either *cis*-element concatemer was highly Tv4-specific; ectopic expression was not observed in the VNC, and was found in only a few cells in the brain (for *6xHD-A-nEYFP*) or late L3 eye imaginal disc (for *4xMad-D-nEYFP*) (**Figs 4B, 4D and S5**). We also tested tetrameric concatemers of other regions from the Tv4-enhancer, but these failed to generate Tv4 neuron expression (**Fig 4C, 4E and 4F**). Thus, even though the short *cis*-elements containing HD-A and Mad-D have distinct sequences and are both required in the native Tv4 enhancer, each *cis*-element contains sufficient sequence information for Tv4 neuron expression.

**Fig 5:**
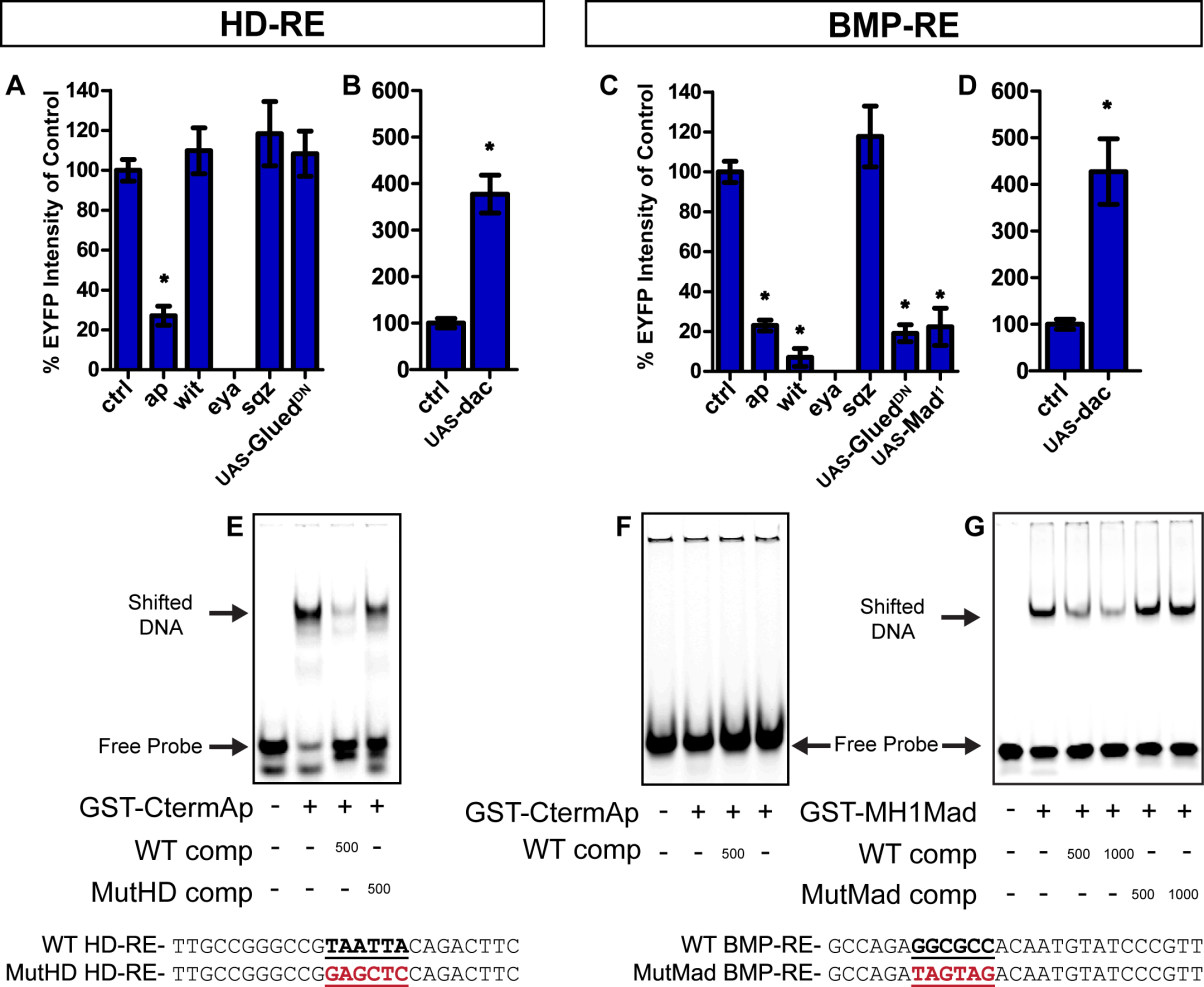
Genetic and biochemical analysis of the HD-RE and BMP-RE concatemers. (**A,B**) HD-RE (*6xHD-A-nEYFP*) and (**C,D**) BMP-RE (*4xMad-D-nEYFP*) reporter expression in the genotypes shown. We show the relative intensity of reporter expression as the % of the mean of the genetic control. (**A**) Expression of HD-RE is reduced in *ap* mutants and eliminated in *eya* mutants, but not altered in *wit* or *sqz* mutants. Expression of *UAS-Glued* ^*DN*^ (retrograde traffic blocker) using *ap* ^*GAL4*^ had no effect on HD-RE. (**B**) *UAS-dac* overexpression (by *OK6-GAL4*) strongly increased HD-RE expression in Tv4 neurons. (**C**) Expression of BMP-RE is reduced in *ap*, *wit* and *eya* mutants. *BMP-RE* expression was also eliminated by overexpression of *UAS-Mad* ^*1*^ (DNA-binding defective Mad) or *UAS-Glued* ^*DN*^ (retrograde trafficking blocker) using *ap* ^*GAL4*^. (**D**) *UAS-dac* overexpression increased *BMP-RE* expression in Tv4 neurons. (**E-G**) EMSA studies show sequence-specific binding of Apterous to the HD-A motif of HD-RE and of Mad to the Mad-D motif of BMP-RE. (**E**) HD-RE labeled probes (sequences shown below as WT HD-RE) are shifted in the presence of recombinant GST-CtermAp (C-terminal half of Apterous containing the Homeodomain) and efficiently out-competed by wildtype unlabeled probe (WT comp). The binding is not outcompeted by unlabeled HD-RE with a mutated HD-A motif (MutHD HD-RE). The number under each lane indicates the stoichiometric ratio between unlabeled and labeled probe. (**F**) The BMP-RE is not shifted in the presence of GST-CtermAp, indicating a lack of Ap binding. (**G**) The BMP-RE is shifted when presented with GST-MH1-Mad (that contains the DNA-binding domain). The band shift is out-competed by increasing amounts of unlabeled wild-type probe (WT comp). Competition is not observed when unlabelled probe with a mutated putative Mad-binding site (MutMad comp) is added. Data in A-C represented as mean±SEM. n = 10–20 animals per genotype and compared using one-way ANOVA with Tukey HSD *post-hoc* test. * = p<0.05 compared to control. **Genotypes.** *[cis-element]* in the following refers to either *6xHD-A-nEYFP* or *4xMad-D-nEYFP*. **(A,C) *ctrl*** *(Tv* ^*[cis-element]*^ */+)*. ***ap***, *(ap* ^*GAL4*^ */ap* ^*P44*^ *; Tv* ^*[cis-element]*^ */+)*. ***wit*** *(Tv* ^*[cis-element]*^,*wit* ^*A12*^ */ wit* ^*B11*^ *)*. ***sqz*** *(Tv* ^*[cis-element]*^,*sqz* ^*ie*^ */sqz* ^*ie*^ *)*. ***eya*** *(eya* ^*Cli-IID*^ */eya* ^*D1*^ *; Tv* ^*[cis-element]*^ */+)*. ***UAS-Mad*** ^***1***^ (*ap* ^*GAL4*^ */UAS-Mad* ^*1*^ *; Tv* ^*[cis-element]*^/*/UAS-Mad* ^*1*^). ***UAS-Glued*** ^***DN***^ (*ap* ^*GAL4*^ */UAS-Glued* ^*DN*^ *; Tv* ^*[cis-element]*^/*+*) **(B,D) *Ctrl*** (*OK6-GAL4* ***/+;*** *Tv* ^*[cis-element]*^ */* ***+*). *UAS-dac*** (*OK6-GAL4* ***/+;*** *Tv* ^*[cis-element]*^ */UAS-dac*).

The Tv4-specific reporter expression of each concatemer provides us with the tools to determine which transcriptional regulators act at each *cis*-element. First, we examined BMP-dependence, as Mad motifs are present in both *cis*-elements. *6xHD-A-nEYFP* was not affected in *wit* null mutants, or after blockade of retrograde BMP signaling, using *ap* ^*GAL4*^ to drive *UAS-Glued* ^*Δ84*^, a truncated allele of *p150* ^*Glued*^ that blocks dynein-dependent retrograde transport, nuclear pMad accumulation and FMRFa expression [13] (**Fig 5A**). In contrast, *4xMad-D-nEYFP* expression was severely reduced in *wit* nulls and *UAS-Glued* ^*Δ84*^ (**Fig 5C**). Overexpression of Mad^1^ (*UAS-Mad* ^*1*^) also eliminated *4xMad-D-nEYFP* expression (**Fig 5C**); Mad^1^ cannot bind DNA but it is phosphorylated, couples to Medea and accumulates in the nucleus normally [58]. We tested the sequence-specificity of Mad binding to the Mad-D motif by electrophoretic mobility shift assay (EMSA) (**Fig 5G**). Purified GST-MH1-Mad (comprising the DNA-binding MH1 domain) band shifted a Mad-D region DNA probe in a GGCGCC sequence-specific manner (**Fig 5G**). Thus, the Mad-D sequence is necessary for activity of the Tv4-enhancer, exhibits BMP-dependent expression as a concatemer, and binds Mad in a sequence-specific manner. Henceforth, we termed this *cis*-element the BMP-Response Element (BMP-RE).

In *ap* mutants, *6xHD-A-nEYFP* was significantly down-regulated to 27% of controls (**Fig 5A**). This was expected due to the consensus Ap-binding sequence in HD-A, and the previous biochemical evidence for Ap binding to this motif [42], as well as the importance of the HD-A to Tv4-enhancer activity. EMSA analysis supported this; GST-CtermAp (the C terminal half of Ap that includes the homeodomain but excludes the LIM domains) band-shifted an HD-A sequence DNA probe in a TAATTA sequence specific manner (**Fig 5E**). Unexpectedly, *4xMad-D-nEYFP* was also reduced to 23% of control intensity in *ap* mutants (**Fig 5D**), in spite of the lack of a putative Ap binding site. GST-CtermAp failed to band shift the BMP-RE sequence. Thus, we postulate that the genetic regulation of the BMP-RE by Apterous is likely mediated via Ap-dependent activation of another transcription factor **(Fig 5F)**. We henceforth term the HD-A *cis*-element the Homeodomain-Response Element (HD-RE).

We examined HD-RE and BMP-RE responsiveness to *dac* and *eya*. Both *cis*-elements are eliminated in *eya* mutants (**Fig 5A and 5E**) and upregulated by ~400% by *dac* overexpression in Tv4 neurons (**Fig 5B and 5D**). Thus, both co-regulators coordinately regulate *trans*-activation from both *cis*-elements. We conclude that Ap and Mad are recruited to the native Tv4-enhancer at distinct *cis*-elements; the Ap-recruiting HD-RE and the Mad-recruiting BMP-RE. While this may explain the combinatorial requirement for both *cis*-elements, our concatemer analysis shows that Tv4-specificity does not necessarily emerge from it being the point of intersection of the partially overlapping spatial activities of these two *cis*-elements. Instead, each *cis*-element independently encodes sufficient information for Tv4-specific expression. This is not explained solely by the activities of Ap or BMP, as these are present in other non-overlapping neuronal populations. Thus, Tv4-specificity presumably requires additional unknown inputs acting at each of these *cis*-elements. For the BMP-RE, this is perhaps an Ap-dependent transcription factor, as Ap is required for BMP-RE reporter activity but does not bind.

### BMP-signaling and Seven up independently coordinate the timing of *FMRFa* initiation

The HD-RE and BMP-RE both encode the same spatial information. Ap acts as a central coordinator of the activity of both *cis*-elements. BMP-signaling acts via Smads at a BMP-RE *cis*-element, yet it also implicitly carries with it a temporal-encoding facet; the requirement for BMP-driven Smads ensures that FMRFa expression is inevitably tied to target contact in late Stg. 17 embryos. This led us to test the following model: The HD-RE is activated after Tv4 neuron birth, but its weak activity is not sufficient to initiate FMRFa expression at this early time. Once the target is contacted and BMP signaling is initiated, the BMP-RE becomes activated, and the combined activities of the HD-RE and the BMP-RE become sufficient to initiate *FMRFa trans*-activation.

**Fig 6:**
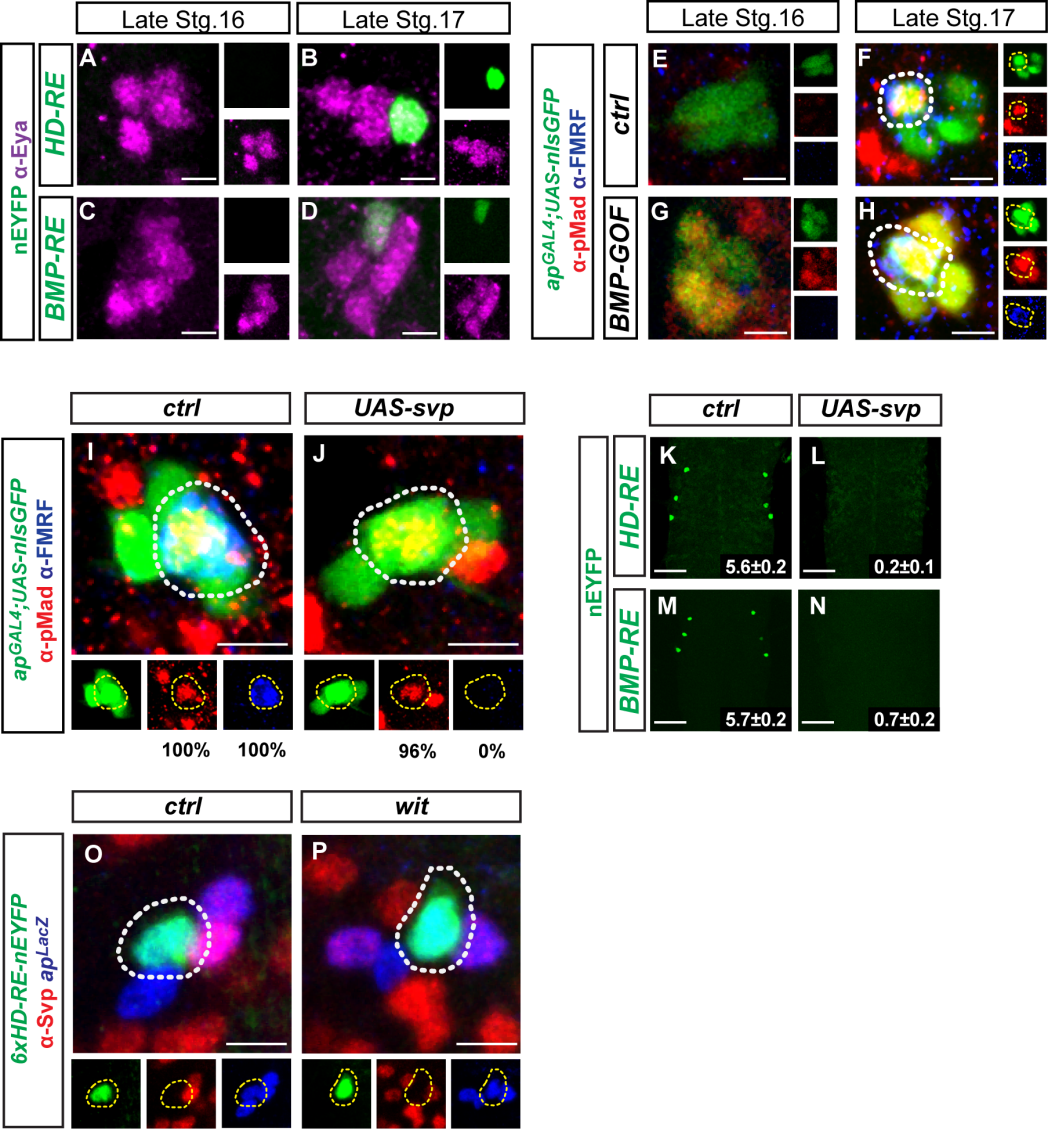
Svp represses *FMRF* via the HD-RE and BMP-RE prior to target contact. (**A-D**) The HD-RE (*6xHD-A-nEYFP)* and BMP-RE (*4xMad-D-nEYFP*) reporters are not expressed at late Stg. 16 (during Svp expression) but become expressed by late Stg. 17 (after mouth hook appearance). (**E-H**) Precocious, robust activation of BMP (shown by pMad immunoreactivity, red) is seen by Stg. 16 in Tv neurons (green), using *ap* ^*GAL4*^ to drive *UAS-tkv* ^*Act*^,*UAS-sax* ^*Act*^ and *UAS-myc*::*mad* transgenes (G). However, even when BMP is precociously activated, FMRFa expression (blue) is not detected until late Stg. 17, its normal initiation time (F,H). Tv4 neurons are indicated by dotted circles. (**I,J**) Maintaining *UAS-svp* expression using *ap* ^*GAL4*^ results in total loss of FMRFa immunoreactivity in all Tv4 neurons, here shown at early L1. Also, pMad accumulation is unaffected in Tv4 neurons in these animals; numbers under insets represent fraction of Tv clusters with pMad or FMRFa expressing cells (n = 30 and 48 for controls and *UAS-svp*, respectively.) (**K-N**) Expression of HD-RE and BMP-RE reporters is strongly reduced by early L1 larvae when *UAS-svp* is overexpressed. Numbers in lower right corner indicate the mean number of nEYFP cells per animal ± SEM (n = 10–16 animals per group, p<10^−3^ two-tailed *t*-test between experimental and control for each reporter). (**O,P**) Svp expression is not detectable in early L1 larvae in control or *wit* mutant animals. Thus, BMP signaling is not required to downregulate Svp expression. The *HD-RE* reporter is used to identify the Tv4 neuron in these *wit* mutants. Tv4 neurons are indicated by dotted circles. Svp immunoreactivity can be detected in Tv2 and Tv3 neurons. **Genotypes**: (**A-D**) Concatemerized *cis*-elements *[cis-element]* (*6xHD-A-nEYFP* or *4xMad-D-nEYFP*) were analyzed as homozygotes. (**E-F**) **BMP gain of function** (*ap* ^*GAL4*^ */+;+/+* vs. *ap* ^*GAL4*^ */UAS-tkv* ^*Act*^,*UAS-sax* ^*Act*^ *;+/UAS-myc*::*mad*). (**I-N**) ***Svp* gain of function** (*ap* ^*GAL4*^ */+;+/+* vs. *ap* ^*GAL4*^ */+;+/UAS-svp)* (*ap* ^*GAL4*^ */+; Tv* ^*[cis-element]*^ */+* vs. *ap* ^*GAL4*^ */+; Tv* ^*[cis-element]*^ */UAS-svp)*. (**O,P**) **BMP loss of function** (*ap* ^*lacZ*^ */+;wit* ^*A12*^ */ Tv* ^*6xHD-A-nEYFP*^ *+* vs. *ap* ^*lacZ*^ */+;wit* ^*A12*^ */ wit* ^*A12*^,*Tv* ^*6xHD-A-nEYFP*^).

This model predicts that the HD-RE (*6xHD-A-nEYFP*) would initiate reporter expression prior to target contact because all known regulators of the HD-RE are present before target contact, and the *cis*-element's activity does not require target-derived BMP-signaling. In contrast, the BMP-RE (*4xMad-D-nEYFP*) would initiate only after target contact because it requires BMP-signaling for its activity. Upon testing this, we unexpectedly found that the HD-RE initiates reporter activity at the same time as BMP-RE and *FMRFa*, at late Stg. 17 (**Fig 6A–6D**). This paradoxical observation cannot be explained by another retrograde signal acting at the HD-RE, as *UAS-Glued* ^*Δ84*^ expression did not affect HD-RE reporter activity (**Fig 5A**). Thus, we postulated that the HD-RE must be responsive to a novel timer that is separate to, but coincidental with, target contact. We reasoned that the existence of a second timer would be further supported if precocious BMP activity in Tv4 neurons could not initiate early *FMRFa* expression, prior to the normal time of target contact. We tested this using *ap* ^*GAL4*^ to drive excess Mad (*UAS-myc*::*mad*) that was phosphorylated by co-expression of *UAS-Tkv* ^*Act*^ and *UAS-Sax* ^*Act*^ [13]. This generated high pMad immunoreactivity in Tv neurons by Stg. 16, prior to target contact (**Fig 6E–6H**). Remarkably, this had no effect on the initiation time of FMRFa. It initiated expression at its normal time at late Stg. 17 (**Fig 6F and 6H**), and failed to activate precociously at late Stg. 16 (n = 42 Tv clusters each for control and experimental) or even mid Stg. 17 (n = 48 Tv clusters for control and experimental) (**Fig 6G**). Thus, *FMRFa* does not simply 'await' target contact and BMP-dependent activation, as is generally assumed for target-dependent gene expression, but its expression is somehow prevented prior to target contact.

Towards identifying a second putative timer, we tested a possible role for the nuclear receptor *seven up (svp)*. *Svp* is required early in the NB5-6T neuroblast lineage (that gives rise to Tv neurons) as a switching factor that triggers the *hunchback* to *Kruppel* temporal transcription factor transition, in part through downregulating *hunchback* expression by an unknown mechanism [59, 60]. A second pulse of Svp expression occurs later in this neuroblast at the time of Tv neuron generation. Its expression is initially retained in all newly born Tv neurons, but then becomes downregulated in Tv1 neurons by Stg. 16, and in Tv4 neurons by early Stg. 17 [60]. This second pulse is required for the appropriate diversification of Tv1-4 neuron subtypes. Lineage confusion amongst Tv neurons is observed in the few Tv neuronal clusters that are generated in *svp* nulls. For example, the Tv4 neuron is not generated, supernumerary Nplp1 Tv1 neurons are produced, and transcription factor expression profiles suggest that many Tv neurons have mixed identities. The authors concluded that *svp* acts during Tv neuron lineage progression to generate diversity amongst Tv1-Tv4 neurons [60].

The coincidental timing of Svp downregulation and FMRFa initiation was intriguing, and prompted us to test the hypothesis that Svp acts in postmitotic Tv4 neurons to repress *FMRFa* up to the time of target contact. First, we tested the effect of maintaining Svp expression beyond its normal time of downregulation (at Stg. 17), by driving *UAS-svp* from *ap* ^*GAL4*^. This was previously shown to prevent expression of Dimmed, Nplp1 and FMRF in Tv1 and/or Tv4 neurons [60]. In early L1 larvae, we found that this eliminated FMRFa, HD-RE and BMP-RE reporter expression (**Fig 6K–6N**), but did not block pMad accumulation in Tv4 nuclei (**Fig 6I and 6J**).

This overlap of pMad and *ap* ^*GAL4*^, *UAS-nGFP* allowed us to uniquely identify Tv4 neurons when *UAS-svp* is overexpressed, in spite of loss of FMRFa. This allowed us to examine if the expression of essential transcriptional regulators of *FMRFa* were downregulated in Tv4 neurons. We found that expression of all the confirmed regulators of *FMRFa* were expressed normally, including pMad, Dac, Eya and *ap (ap* ^*GAL4*^,*UAS-nGFP*) (Figs 6 and S6). Thus, unique Tv4 neuron identity and retrograde BMP signaling were unaffected by persistent Svp. This shows that maintained Svp expression blocks terminal differentiation of FMRFa expression in Tv4 neurons, rather than prevents the generation, axon targeting or BMP activation of Tv4 neurons. The coincidence of Svp downregulation with Tv4 neuron target contact suggested that BMP activation might be the trigger for Svp downregulation. We tested this by examining Svp immunoreactivity in *wit* mutants. Using *6xHD-A-nEYFP* to identify Tv4 neurons in *wit* mutants, we found that Svp immunoreactivity was downregulated at its normal time in the absence of BMP signaling (**Fig 6O and 6P**), indicating that BMP activation does not downregulate Svp.

We next examined whether the effect of Svp on the HD-RE and BMP-RE is mediated by direct Svp binding. Previous characterization including high throughput studies had identified a core Svp bipartite motif of two GGTCA half-sites separated by a short spacer [61–63]. We first confirmed that full-length recombinant Svp can shift a labeled DNA probe containing the characterized DR1 bipartite Svp binding sites in a sequence specific manner, using established conditions [62] (S7A Fig). Next, we found a near-consensus Svp half-site in the HD-RE and an adjacent half-site 4 bp away and outside the HD-RE (S7B Fig) Using an extended probe that included both of these candidate half sites, we examined whether recombinant Svp could bind this DNA sequence probe, in a sequence specific manner. A weak band shift of this extended HD-RE region was observed in the presence of recombinant Svp, using the same conditions as used for the DR1 element (S7B Fig Lanes10-15). Cold competitor sequences with mutated putative Svp binding sites competed as efficiently as wild-type competitors. Thus, Svp does not bind HD-RE in a sequence-specific manner. Addition of poly-dI-dC strongly reduced the HD-RE band shift (S7B Fig Lanes 16–19). The BMP-RE lacked any putative Svp binding sites and did not show any appreciable band shift in the presence of recombinant Svp (S7C Fig) Collectively, these data fail to support a model in which the HD-RE or BMP-RE are regulated by Svp through direct binding.

These results suggest that Svp gates *FMRFa* initiation by indirectly preventing HD-RE and BMP-RE activity prior to target contact. Immediately prior to target contact, Svp expression is downregulated, which de-represses the HD-RE and BMP-RE. At the time of target contact, BMP signaling can then activate expression. This model would predict that loss of *svp* should activate *FMRFa* prematurely. Testing this directly is complicated by two factors. Tv4 neurons are not generated in *svp* nulls, and *FMRFa* initiation requires BMP activation that is temporally coincident with Svp downregulation. To test our hypothesis in a way that avoids these confounding factors, we examined the timing of HD-RE (*6xHD-A-EYFP*) initiation in strong *svp* hypomorphs, because this *cis*-element is BMP-insensitive but is driven by Ap, Dac, and Eya that are all expressed from the birth of the Tv4 neuron. We found that *svp* ^*1*^ */svp* ^*2*^ generates a normal number of correctly specified Tv neurons, including a single Nplp1-expressing Tv1 neuron and a single FMRFa-expressing Tv4 neuron (S6E Fig), yet is a strong enough allelic combination to generate other *svp* lineage phenotypes [64].

We tested the initiation time of HD-RE (*6xHD-A-nEYFP*) at numerous developmental stages in control and *svp* mutant embryos: Early Stg. 17, when the gut is beginning to fold but is not yet showing great complexity; Mid Stg. 17, at a time before the trachea starts to air fill; Air-filled trachea (AFT) stage, when the trachea is filling or filled but before mouth hooks form; Air-filled trachea and mouth hooks (AFT/MH), when both structures are well developed immediately prior to hatching. In controls, heterozygotic *6xHD-A-*nEYFP was expressed in 28% of Tv4 neurons by AFT/MH and not at any stage prior. In contrast, in *svp* ^*1*^ */svp* ^*2*^ mutants, we observed reporter expression in 61% of Tv4 neurons at AFT/MH, also in 35% of Tv4 neurons at AFT, and in 14% of Tv4 neurons at mid Stg17 (Table 1). This shows that the reporter is initiated at an earlier timepoint in *svp* hypomorphs, and that its expression is more robust that in controls by late Stg. 17. This premature initiation time was *svp* dose-dependent, since *svp* ^*1*^ */+* heterozygous reporter expression was observed in 36% of Tv4 neurons at AFT/MH and prematurely in 26% of Tv4 neurons at AFT.

**Table 1:** Reduction in *svp* gene dosage results in premature *6xHD-A-nEYFP* reporter expression.

Embryonic Stage	Early Stg. 17	Mid Stg. 17	AFT	AFT/MH
***+/+***	N/A	**0%** (0/114)	**0%** (0/18)	**28%** (17/60)
***svp*** ^***1***^ ***/+***	**0%** (0/6)	**0%** (0/45)	**26%** (9/34)	**36%** (22/61)
***svp*** ^***1***^ ***/svp*** ^***2***^	**0%** (0/51)	**14%** (6/43)	**35%** (7/20)	**61%** (35/57)

As *svp* dosage is reduced from controls (+/+), to heterozygotes (*svp* ^*1*^ */+*), to hypomorphs (*svp* ^*1*^ */svp* ^*2*^), we observed increasingly premature reporter expression, indicative of progressive de-repression of its activity. Data in bold presented as the % of total Eya-positive, Tv-clusters expressing *6xHD-RE-nEYFP* at the embryonic stages shown. In brackets, we show the fraction of the total number of Tv clusters that exhibit nEYFP reporter expression.

We conclude that two independent timers together regulate the timing of *FMRFa* initiation. The first timer is Svp that acts as an intrinsic repressor that prevents HD-RE and BMP-RE activity prior to target contact. Downregulation of Svp immediately prior to target contact de-represses HD-RE and BMP-RE activity. The second timer is target-activated BMP signaling that directly activates *FMRFa* via Mad binding to the BMP-RE *cis*-element. Interestingly, although these two timing events are temporally coincidental, we find no evidence for a cross-regulatory genetic hand-over from one timer to the other.

## Discussion

Target-dependent gene expression in many neurons is initiated upon contact of axons and/or dendrites with their target cell(s), but the underlying gene regulatory mechanisms are largely unexplored [3, 4]. Here, we examined these gene regulatory mechanisms, using initiation of the *FMRFa* gene in Tv4 neurons by target-dependent BMP-signaling as a model. We uncover key *cis*-regulatory sequences in a Tv4-enhancer of the *FMRFa* gene that integrate the necessary and combinatorially sufficient inputs of Ap, Dac, Eya and BMP-activity to generate *FMRFa* expression in Tv4 neurons upon target contact. These studies show that BMP-signaling contributes through Smad binding at an essential *cis*-element, and reveals surprising complexity in the integration of intrinsic and extrinsic inputs at the FMRFa enhancer. In addition, we provide evidence to support an hypothesis that target-dependent genes are repressed prior to target contact (in this case by *svp*), rather than simply awaiting activation. These genes become de-repressed around the time of target contact in order for the target-derived signal to be able to directly activate the gene's expression.

### *cis*-regulation of spatiotemporal *FMRFa* expression

We aimed to identify the *cis*-regulatory sequences and core regulatory mechanisms through which a genetically identified set of regulatory inputs determines the Tv4-specific expression of *FMRFa* upon target-derived BMP-signaling. We found that activity of the Tv4-enhancer requires the sequence-specific regulators Ap and BMP-activated Smads and the co-regulators Dac and Eya. As these inputs are all genetically necessary and combinatorially sufficient for *FMRFa* expression [13, 41], we focused on the mechanisms through which these critical regulators specify *FMRFa* expression. We found that Ap and BMP-activated Smads bind directly at the Tv4-enhancer, but that their binding is parsed onto two distinct and essential *cis*-elements, the HD-RE and BMP-RE, respectively. Thus, the combinatorial requirement for Ap and BMP appears to be conferred by the integration of both essential *cis*-elements. This indicates that BMP-signaling acts directly at the *FMRFa* enhancer. We propose that BMP-signaling forms part of the combinatorial code of transcriptional inputs that together specify *FMRFa* gene expression, as opposed to triggering the transcriptional activity of a transcriptional complex that is pre-established at the *FMRFa* enhancer.

We identified two levels of regulatory coordination between the two *cis*-elements. Ap binds the HD-RE and is necessary for its activity, but Ap is also required indirectly for BMP-RE activity without direct binding. We postulate that Ap likely regulates the expression of an unknown transcription factor that binds and activates the BMP-RE, but verification of this model awaits the identification of Ap-dependent transcription factors acting at the BMP-RE. Also, we found that Dac and Eya are both important co-regulators that mediate the activities of both *cis*-elements. Eya in part mediates this effect by regulation of BMP-activity in Tv4 neurons and does not contribute to ectopic *FMRFa* expression when Ap, Dac and BMP signaling are present [41]. In contrast, we show here that Dac is a potent amplifier of HD-RE, BMP-RE and *FMRFa* expression in late embryos and early larvae. Dac does not appear to be required for the native low-level *FMRFa* expression in the late embryo and early L1 larval stage. However, Dac becomes essential for the high level expression of *FMRFa* thereafter [37], as well as for generation of ectopic *FMRFa* expression induced by Ap and BMP-signaling in other neurons. Although the function of Dac in gene regulation is still ambiguous in most contexts, it is generally viewed as a co-regulator that recruits histone modifying complexes and the mediator complex [65–68]. Thus, we postulate that Dac may promote a chromatin state that facilitates high-level transcriptional activation downstream of Ap/Smad engagement of *FMRFa cis*-regulatory sequences. Such a model will require detailed analysis, and likely also identification of other transcription factors acting at the HD-RE and BMP-RE *cis*-elements that may be required for recruitment of Dac. In this light, it is interesting to note that DNA-bound vertebrate Smad4 has been shown to recruit Dach1, which acts in that context as a co-repressor that recruits the nuclear receptor co-repressor (N-CoR), that in turn recruits histone deacetylases [69, 70].

Towards identifying the information that each *cis*-element contributes to overall Tv4-enhancer activities, we generated concatemers of the HD-RE and BMP-RE *cis*-elements. Unexpectedly, both HD-RE and BMP-RE concatemers independently generated the same spatiotemporal pattern as the full Tv4-enhancer. Thus, taken together with our finding that both *cis*-elements are required in the native Tv4-enhancer context, we conclude that the HD-RE and BMP-RE are low activity *cis*-elements required in combination for *FMRFa* expression but that encode the same spatiotemporal information from distinct inputs. These results were not expected, and dispel the simplest prediction that the HD-RE receives cell-specific transcription factor input contributing spatial information, while the BMP-RE receives the extrinsic BMP input contributing temporal information. Such a model would have been in line with evidence from examination of other enhancers in which the correct spatiotemporal expression is generated by combining the activities of distinct spatial and temporal encoding *cis*-elements [69–76]. However, the Tv4-enhancer does not appear to act as an integrator of differential spatial and temporal inputs encoded via these two *cis*-elements, as both *cis*-elements encode full spatiotemporal information from their respective developmental inputs.

It is unclear why two *cis*-elements encoding the same spatiotemporal information are utilized, when either one could conceivably function alone. One rationale could derive from the small amount of ectopic, non-overlapping expression that is generated by the HD-RE or BMP-RE concatemers. Such non-overlapping ectopic expression may indicate that these *cis*-elements have low-level activity so as to restrict *FMRFa* activation only to cells where both *cis*-elements are activated. Indeed, attenuation of *cis*-element activity to restrict target gene expression has been demonstrated, via reduced transcription factor affinity or by inclusion of repressive elements [77, 78]. Another mechanism may be related to the ability of multiple weak *cis*-elements to generate robust and specific gene expression. For example, shadow enhancers are *cis*-elements with similar spatiotemporal outputs that act redundantly (to varying degrees) in normal conditions, but are required together for robust output in adverse conditions [79, 80]. Also, the addition of increasing numbers of redundant but individually weak *cis*-elements was shown to increase the robustness of Sonic hedgehog gene expression in different mouse tissues [81]. Moreover, robust and specific expression can be achieved by the accumulation of multiple low activity *cis*-elements; multiple Ultrabithorax binding sites are required together for spatially-restricted repression of *spalt* in the *Drosophila* haltere [82], and multiple weak Ultrabithorax-Extradenticle binding sites drive *shaven baby* in *Drosophila* epidermal tricomes [83]. Thus, the use of two discrete low activity *cis*-elements that generate the same spatiotemporal output from different developmental inputs may offer a solution for integrating all the appropriate spatial and temporal inputs into robust, exquisitely specific activity in only 6 neurons of the nervous system.

Our analysis raises some unresolved questions. First, the specificity of the HD-RE and BMP-RE *cis*-elements remains unexplained, as Ap and BMP activity cannot alone explain HD-RE and BMP-RE spatiotemporal expression. Both regulators are active in many other neurons, yet the HD-RE and BMP-RE concatemers are not expressed in these neurons. Thus, unknown regulators must act with Ap or Smads at these *cis*-elements. We aim to identify those transcription factors in ongoing screens, because models that account for the Tv4-specificity of either *cis*-element will require incorporation of those transcription factors' activities. Second, the low conservation region between the HD-RE and BMP-RE contains sequence-specific information that is critical for enhancer activity. At this time, no identified transcriptional regulator has been predicted or shown to act at this region. Future analysis of this region awaits the identification of transcription factors that may act within this region. Finally, deletion or point mutagenesis of sequences 3' of the BMP-RE identify other regions that contribute to overall expression level. However, because none of these regions were found to be absolutely critical for enhancer activity in our assays, we did not focus on these in this study, and their precise contribution remains untested.

### Pre-target contact repression of a target-dependent gene

The developmental initiation of target-dependent genes in neurons requires target contact and target-derived signaling, making it reasonable to assume that these genes simply wait to be activated prior to target contact. However, our seemingly paradoxical results regarding the timing of *FMRFa* activation lead us to a novel model wherein target-dependent genes are repressed prior to target contact: First, the BMP/target-insensitive HD-RE *cis*-element initiated expression at the same time as the BMP-RE *cis*-element and *FMRFa* itself. Second, precocious BMP activation failed to initiate *FMRFa* at an earlier timepoint. These data suggested that the HD-RE *cis*-element responds to another timer that prevents BMP-dependent *FMRFa* activation prior to target contact. We considered two possibilities: First, an unknown and necessary regulator is not expressed until the time of target contact. Second, a repressor is active prior to target contact. Our evidence supports the second, novel model. Previous work had shown that Svp is downregulated immediately prior to target contact [60]. Here, we found that this downregulation is required to de-repress the Tv4-enhancer via both the HD-RE and BMP-RE, as maintained Svp expression blocks the induction of HD-RE, BMP-RE and *FMRFa*. Moreover, we show that HD-RE *cis*-element expression initiates at increasingly earlier time points as *svp* dosage is reduced. This demonstrates that Svp expression level gates the initiation time of this *cis*-element. The mechanism by which Svp represses *FMRFa* is unknown; it does not alter the expression of known *FMRFa* regulators, and EMSA analysis did not support a role for direct Svp-binding to the HD-RE or BMP-RE *cis*-elements. Possible mechanisms include regulation of the expression of unidentified essential transcription factors, or direct interference by Svp on the transcriptional activities of transcription factors or chromatin modifiers.

The *seven up* gene is an intriguing factor to play a role in gating the timing of terminal differentiation. In both *Drosophila* and vertebrates, Svp (vertebrate COUP-TF I/II) is a temporal switching factor that mediates transitions in the developmental potential of neuroglial lineages (reviewed by [59, 84]). In *Drosophila*, a transient Svp pulse triggers the *hunchback* to *Kruppel* switch, by repressing *hunchback*, in the neuroblast temporal transcription factor cascade in multiple lineages [64, 85], including in the NB5-6T lineage [60]. Also, in late larvae, a transient pulse of Svp is required to switch neuroblasts from expressing Chinmo to expressing Broad-Complex, which switches the fate and size of neuronal progeny [86]. Svp also acts as a sub-temporal switch to increase the diversity of Tv1-4 neuronal fates generated through the Castor/Grainy head temporal window late in the NB5-6T lineage [60]. Such switching roles are well conserved in vertebrates. The *svp* orthologs COUP-TFI/II are transiently expressed and required to switch numerous progenitor lineages from generating neurons to generating glial cells [87]. In spite of these many characterized switching roles for Svp/COUP-TFI/II, neither the regulation of Svp/COUP-TFI/II pulses nor its downstream molecular actions are well understood in any system.

In conclusion, our work reveals the complex *cis*-regulatory mechanisms of neuronal subtype-specific and target-dependent gene initiation in the context of the target/BMP-dependent induction of FMRFa in Tv4 neurons. Detailed functional analysis of the *cis*-regulatory architecture of other target-dependent neuronal genes will determine whether principles learned here are unique to the *FMRFa* gene, or generalizable to most target-dependent genes.

## Materials and Methods

### Fly genetics

The following strains were used: *sqz* ^*ie*^ and *UAS-sqz* [13]; *UAS-ap*, *ap* ^*RK506*^ (*ap* ^*LacZ*^) [88]; *ap* ^*P44*^ and *ap* ^*md544*^ (*ap* ^*GAL4*^) [89]; *dac* ^*3*^ [90]; *UAS-dac* [91]; *eya* ^*Cli-IID*^ [92]; *eya* ^*D1*^ [93]*; dimm* ^*rev4*^ and *dimm* ^*P1*^ [45]; *grh* ^*IM*^ [94]; *Df(2R)Pcl7B* (*grh* ^*Df*^) [95], *OK6-GAL4*, *wit* ^*A12*^ and *wit* ^*B11*^ [22]; *svp* ^*1*^ and *svp* ^*2*^ [96]; *UAS-Mad* ^*1*^ [58]; *UAS-Glued* ^*Δ84*^ (*UAS-Glued* ^*DN*^) [97]*; UAS-tkv* ^*Act*^ and *UAS-sax* ^*Act*^ [98]; *UAS-myc*::*Mad* [99]; *UAS-svp type I (UAS-svp*) [100]; *UAS-nls*.*EGFPP; Df(2L)Exel7066* (*Dac* ^*Df*^ *)* (Bloomington, IN). Mutants were kept over *CyO*,*Act-GFP TM3*,*Ser*,*Act-GFP* or *CyO*, *twi-GAL4*,*UAS-2xEGFP* or *TM3*,*Sb*,*Ser*,*twi-GAL4*,*UAS-2xEGFP*. *w* ^*1118*^ was used as the control genotype. Flies were maintained at 25°C, 70% humidity.

### Transgene construction

The empty Tv-nEYFP vector was generated from pUASTattB [101] digested with NheI/SpeI and blunted with Klenow fragment. The LoxP and attB sequences from pUASTattB and the multiple cloning site (MCS), HSP70 promoter, Tra nuclear localization signal and SV40-polyA sequences from pHstinger [102] were joined with EYFP from pDUAL-YFH1c [103] using SOE PCR to produce an EcoRV-loxP-MluI-MCS-hsp70 pro-EYFP-*tra*.*nls*-SpeI-SV40polyA-AvrII-attB-ZraI cassette that was digested with EcoRV/ZraI and ligated into the blunted pUASTattB backbone. The Wild type Oregon R Tv4-enhancer was PCR-amplified with EcoRI / XbaI adaptors in the primers, restriction digested and ligated into XbaI/EcoRI digested empty Tv-nEYFP. Nucleotide substitution and deletion mutants were generated by SOE PCR and similarly inserted into EcoRI / XbaI sites. The XbaI and NheI sites were used for the concatemers. Fly transformations were performed by Genetic Services Inc. (Cambridge, MA.) All transgenes were integrated into *attP2* [104]. The Oregon R Tv4-enhancer contains two single nucleotide polymorphisms (SNPs), compared to the reference genome (v4 to v6). Recently sequenced wild *Drosophila* species concur with the Oregon R sequence; thus it is the reference genome that contains atypical SNPs [105]. A summary of all mutations and concatemerization sequences can be found in S1 Table.

### Immunochemistry

Standard protocols were used throughout [106]. *Primary antibodies*: Rabbit and Chicken α-FMRFa C-terminal peptide (1:1000) [39], Rabbit α-FMRFamide (1:2000; T-4757 Peninsula Labs); Chicken α-ß-Gal (1:1000, ab9361, Abcam); Guinea Pig α-Dimm (1:1000) [39]; Mouse α-Eya (1:100; MAb clone 10H6) and Mouse α-Dac (1:2; MAb Dac 2-3clone) (DSHB; Iowa U., Iowa); Rabbit α-pMad (1:100, 41D10, Cell Signaling Technology); Mouse α-Svp (1:50) [60]. *Secondary antibodies*: Donkey anti-Mouse, anti-Chicken, anti-Rabbit, anti-Guinea Pig (H+L) conjugated to DyLight 488, Cy3, Cy5 (1:100, Jackson ImmunoResearch).

### Image and statistical analysis

More than 5 animals were examined for every genotype. Analysis on the 445 bp Tv-enhancers was performed on homozygous reporter lines. Concatemerized *cis*-elements were analyzed as heterozygous reporters. Images were acquired with an Olympus FV1000 confocal microscope with settings that avoided pixel intensity saturation. Fluorescent intensity of individual Tv4 neurons was measured (or from Eya-positive Tv cluster when no Tv4 marker was detectable) in Image J (US National Institutes of Health). Mean pixel intensity for each neuron was measured from summed Z-projection and background fluorescence for subtraction was measured from an adjacent location using the same size circular region of interest. Each datum point of resulting nEYFP intensity was used to calculate mean intensity for a genotype or enhancer variant; each datum point was then represented as a percentage of the mean of the control group. Representative images of Tv neurons being compared in Figs were linear contrast enhanced together in Adobe Photoshop CS5 (Adobe Systems, Mountain View, CA). All statistical analysis and graphing were performed using Prism 5 (GraphPad Software, San Diego, CA). All multiple comparisons were done with One-Way ANOVA and a Tukey *post-hoc* test or Student’s two-tailed *t*-test when there were only two groups. Differences between groups were considered statistically significant when p<0.05. Data are presented as mean ± Standard Error of the Mean (SEM).

### Recombinant transcription factor expression and EMSA

Recombinant GST-CtermAp (LIM domains removed) and GST-MadN (the MH1 domain), were fused to the GST in pGEX6p1 (GE Health), expressed in Rossetta bacteria cells (EMD Millipore, Billerica, MA), purified using Glutathione-Sepharose beads (GE Health), and dialyzed into 20 mM HEPES pH 7.8, 50 mM KCl, 1 mM DTT, and 10% glycerol. Aliquots were stored at -80°C. Full length *svp* Type I cDNA [96] was cloned from *UAS-svp I* [100]. An N-terminus His tag was added to the Svp Type I cDNA and inserted into pGEX6p1. The GST-tagged His::Svp was expressed in Rossetta bacteria cells and purified on Glutathione-Sepharose beads as above, but with the removal of the GST tag using the PreScission Protease kit according to manufacturer’s instructions (GE Health) before concentration and storage as above. Oligonucleotide probes were synthesized and labeled with IRDye 700 by (IDT Inc, Coralville, IA). Gel shift assay for HD-RE or BMP-RE and Apterous binding was performed by incubation (30 min at 21 ^0^C) of 1 μg of GST-CtermAp with 1 μl of 100 nM probe in a 20 -μl reaction buffer (20 mM HEPES pH7.8, 50 mM KCl, 10% glycerol, 0.25 mM EDTA, 0.1 mg/ml BSA, 1 mM DTT). Gel shift assay for BMP-RE and Mad binding was performed by incubating 300 ng of GST-Mad with 1 μl of 50 nM probe in 20 μl reaction buffer (25 mM Tris pH 7.5, 35 mM KCl, 80 mM NaCl, 5 mM MgCl_2_, 3.5 mM DTT, 0.25% Tween 20, 1 μg poly(dI-dC), and 1x Protease Inhibitor cocktail (Roche)). Svp binding to all tested probes was performed by incubating 1 μg of His::Svp with 1 μl of 50 nM probe in 20 μl Svp binding buffer (100 mM KCl, 7.5% glycerol, 20 mM HEPES pH 7.5, 1 mM DTT and 0.1% Nonidet P-40) on ice for 15 min with or without 1 μg of poly(dI-dC) [62]. For competition assays, unlabeled DNA sequences or mutant DNA sequences were incubated with the labeled probes. See Figs for stoichiometric ratio of unlabeled to labeled probe for each EMSA. DNA-protein complexes were resolved on a 4% non-denaturing polyacrylamide gel, and imaged immediately on a Licor Odyssey Imager system (Lincoln, NE.)

## Supporting Information

S1 Fig Expression of the *Tv* ^*WT*^ *-nEYFP* reporter is regulated by a subset of transcription factors known to affect *FMRFa* gene and peptide expression. **(A-F)** Expression of FMRFa and *Tv* ^*WT*^ *-nEYFP* in mutant genotypes. These are representative images for the data shown quantitatively in the bar graph in Fig 2D, that were not shown in Fig 2A, 2B and 2C. **(G-J)** Representative images of the quantitative gain of function data shown in Fig 1 using *OK6-GAL4* to drive *UAS-ap*, *UAS-dimm* or *UAS-dac*. Whole VNCs were imaged through the entirety of their z-axis. Scale bars are 30 μm. Loss of function genotypes: ***ctrl*** *(Tv* ^*WT*^ *-nEYFP)*. ***dimm*** *(dimm* ^*rev4*^ */dimm* ^*P1*^ *; Tv* ^*WT*^ *-nEYFP)*. ***dac*** *(Df(2L)Exel7066/ dac* ^*3*^ *; Tv* ^*WT*^ *-nEYFP)*. ***eya*** *(eya* ^*Cli-IID*^ */eya* ^*D1*^ *; Tv* ^*WT*^ *-nEYFP)*. ***grh*** *(grh* ^*IM*^ */grh* ^*Df*^ *; Tv* ^*WT*^ *-nEYFP)*. ***sqz*** *(Tv* ^*WT*^ *-nEYFP*,*sqz* ^*ie*^ */ Tv* ^*WT*^ *-nEYFP*,*sqz* ^*ie*^ *)*.
(TIF)

S2 Fig Confirmation of *sqz* mutant phenotype in *Tv* ^*WT*^ *-nEYFP* reporter experiments. **(A,B)** Anti-Nplp1 staining in VNC thoracic segment 2 (T2) Tv cluster cells shows the expected supernumerary Nplp1 immunoreactive cells in *sqz* mutants, but not heterozygous controls. Nplp1 cells are marked with asterisks. Scale bars are 5 μm.
(TIF)

S3 Fig Sequence identity of conserved regions of the Tv4-enhancer across 12 *Drosophila* species. Mad sites are highlighted in red, Medea sites in yellow, Homeodomain sites in green throughout this Fig. The only consensus Mad site (MadD) is shown in purple. (**A**) Whole Tv4-enhancer showing conserved HD, Mad and Med regions. (**B**) Sequence identity of HD-A (green), Mad-A (red), Med-A (yellow) across 12 *Drosophila* species (**C**) Sequence identity of Mad-D (purple) across 12 *Drosophila* species. (**D**) Sequence identity of HD-B (green) and Mad-B (red) across 12 *Drosophila* species. (**E**) Sequence identity of HD-C (green) and Mad-C (red) across 12 *Drosophila* species
(PDF)

S4 Fig Tv4-enhancer sequences for 12 *Drosophila* species. Capitalized blue letters denote identity to *D*.*melanogaster* Tv4-enhancer sequence. Thick underline denotes BMP-RE. Double underline denotes HD-RE. Red highlight indicates putative Mad-binding sequence. Magenta highlight indicates putative Mad-binding sequence of Mad-D. Green highlight indicates putative Ap-binding sequence. Yellow highlight indicates putative Medea-binding sequence.
(PDF)

S5 Fig Restricted and non-overlapping ectopic expression of concatemerized BMP-RE and HD-RE reporters. **(A)** Expression of *4xMad-D-nEYFP* in late L3 larvae eye disc. (**B**) Expression of *6xHD-A-nEYFP* in the brain lobes of early L1 larva. Scale bars are 30 μm.
(TIF)

S6 Fig *UAS-svp* gain of function does not affect Eya or Dac expression, and *svp* ^*1*^ */svp* ^*2*^ mutants generate normal Tv clusters. (**A-D**) Maintaining *UAS-svp* expression using *ap* ^*GAL4*^ does not affect Dac or Eya expression by early L1 larval stages (n = 15 Tv4 neurons per group). (**E**) Nplp1 and FMRFa expression are unaffected in the Tv clusters of *svp* ^*1*^ */svp* ^*2*^ animals at late Stg 17. Scale bars represent 5 μm. Dotted circle indicates Tv4 cell. ***Svp* gain of function** (*ap* ^*GAL4*^ */+;+/+* vs. *ap* ^*GAL4*^ */+;+/UAS-svp)*.
(TIF)

S7 Fig EMSA of potential Svp binding to regions of the Tv4-enhancer. (**A**) Positive control EMSA using the previously published DR1 Svp binding site oligonucleotides labeled with IRDye700, subjected to binding with purified recombinant His::SVP. Svp binding sites are underlined. The 20:1 stoichiometric ratio of unlabeled competitor to labeled probe is indicated above the lane number in all gels (20). His::Svp generates a strong band shift of the DR1 sequence that is out-competed by wild-type unlabeled competitor (compare **Lanes 2,3**). Addition of an unlabeled mutated half-Svp site or a non-Svp binding EcR competitor failed to reduce the expected band shift (**Lanes 4,5**). Addition of poly(dI-dC) does not noticeably affect band shifts (**Lanes 6–9**). (**B**) Under the same binding conditions as the DR1 element, addition of His::Svp generated a very weak band shift of the extended HD-RE, containing two putative Svp binding sites that are underlined (**Lanes 10,11**). Competition by wildtype or mutant unlabeled competitors does not alter His::Svp binding to the labeled probe (**Lanes 12–15**). Addition of poly(dI-dC) strongly decreases the intensity of the extended HD-RE band shift (**Lanes 16–19**). (**C**) Addition of His::Svp does not generate appreciable band shift of the BMP-RE (**Lanes 20–31**) in any condition.
(TIF)

S1 Table Summary of Tv4 enhancer sequence mutations used. (DOCX)
