# Supplementary figures and images for: Gene Regulatory Mechanisms Underlying the Spatial and Temporal Regulation of Target-Dependent Gene Expression in Drosophila Neurons
Source: PLoS Genet. 2015 Dec 29;11(12):e1005754. doi: 10.1371/journal.pgen.1005754 (PMC4694770; doi:10.1371/journal.pgen.1005754)

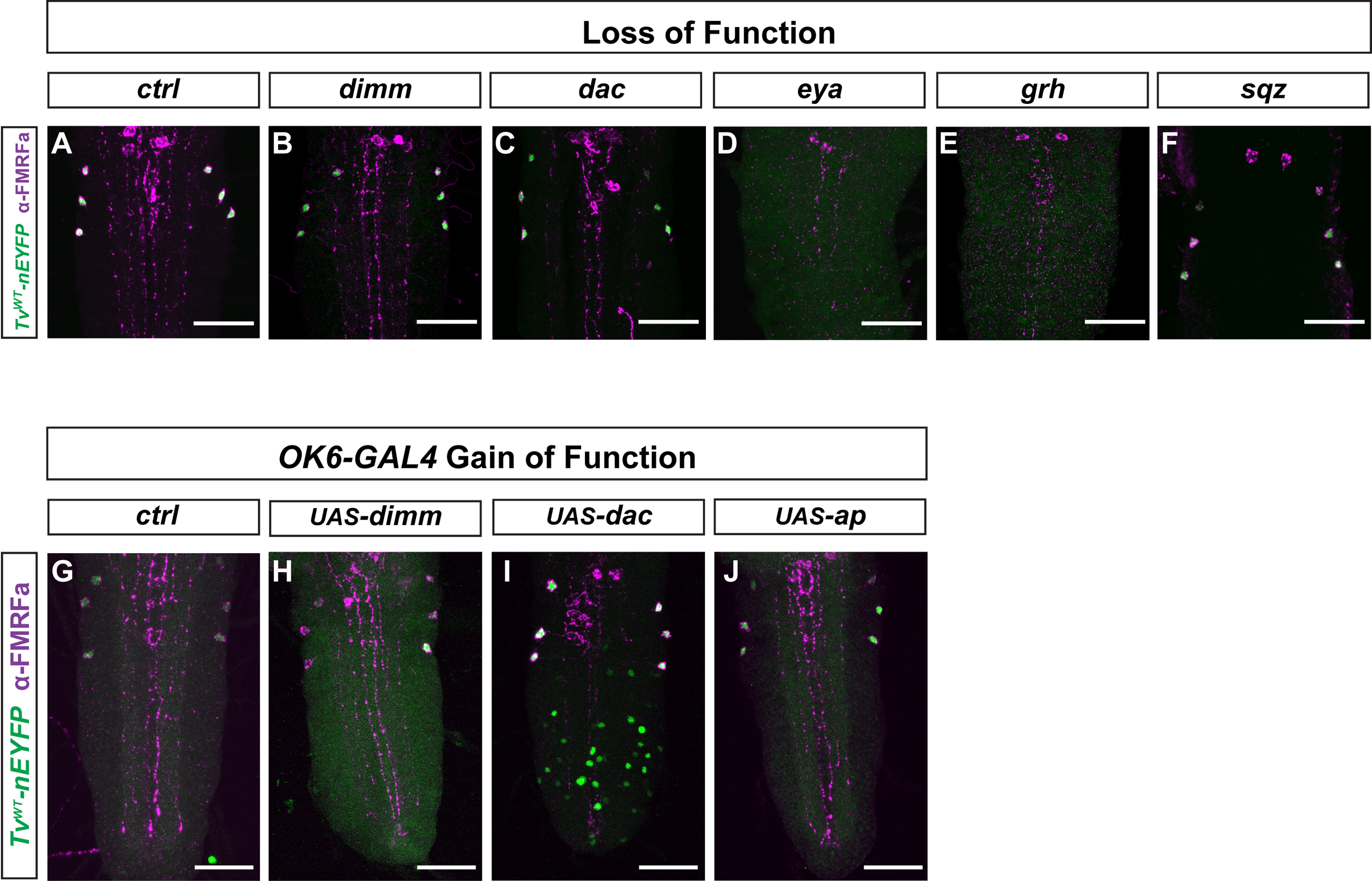

Supplement: S1 Fig — (A-F) Expression of FMRFa and Tv WT -nEYFP in mutant genotypes. These are representative images for the data shown quantitatively in the bar graph in Fig 2D, that were not shown in Fig 2A, 2B and 2C. (G-J) Representative images of the quantitative gain of function data shown in Fig 1 using OK6-GAL4 to drive UAS-ap, UAS-dimm or UAS-dac. Whole VNCs were imaged through the entirety of their z-axis. Scale bars are 30 μm. Loss of function genotypes: ctrl (Tv WT -nEYFP). dimm (dimm rev4 /dimm P1 ; Tv WT -nEYFP). dac (Df(2L)Exel7066/ dac 3 ; Tv WT -nEYFP). eya (eya Cli-IID /eya D1 ; Tv WT -nEYFP). grh (grh IM /grh Df ; Tv WT -nEYFP). sqz (Tv WT -nEYFP,sqz ie / Tv WT -nEYFP,sqz ie ). (TIF) [file pgen.1005754.s001.tif]

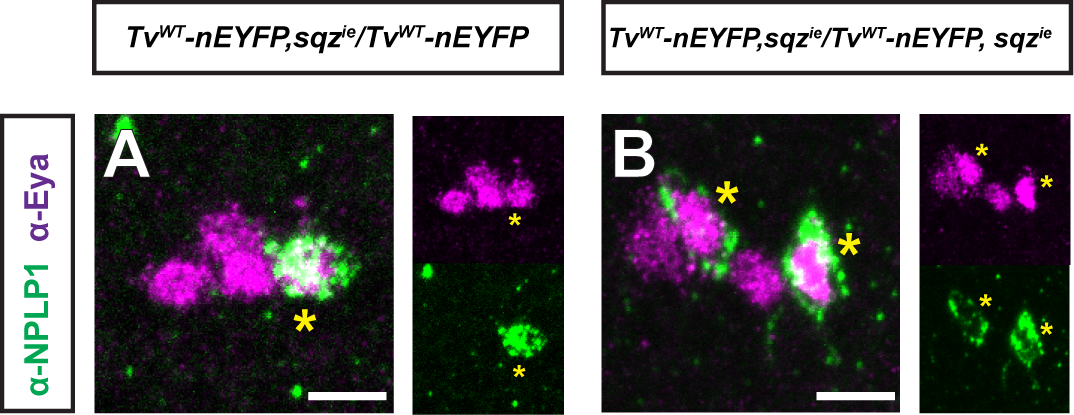

Supplement: S2 Fig — (A,B) Anti-Nplp1 staining in VNC thoracic segment 2 (T2) Tv cluster cells shows the expected supernumerary Nplp1 immunoreactive cells in sqz mutants, but not heterozygous controls. Nplp1 cells are marked with asterisks. Scale bars are 5 μm. (TIF) [file pgen.1005754.s002.tif]

**A**

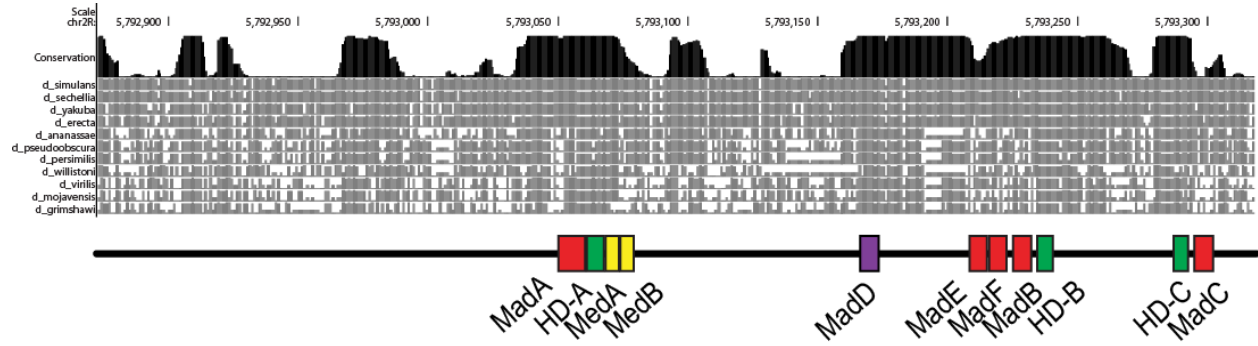

**B**

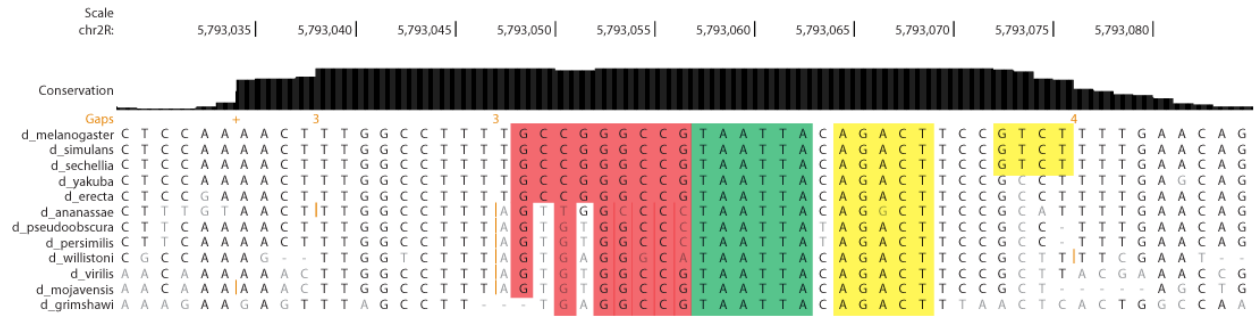

**C**

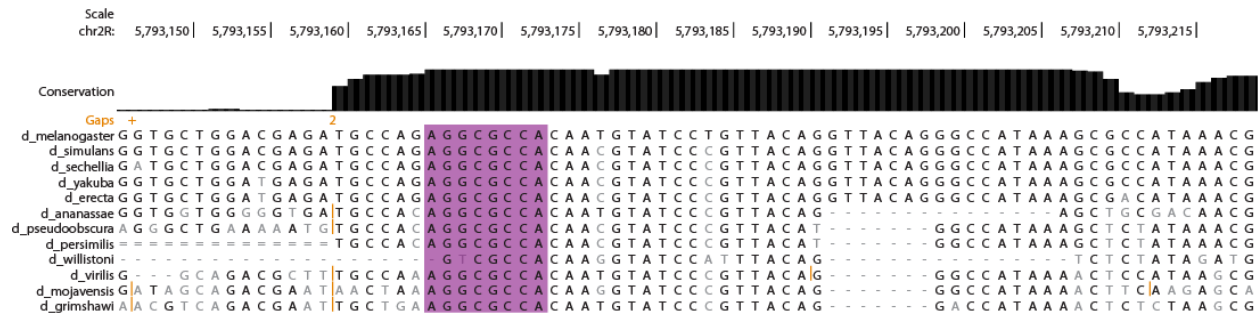

D

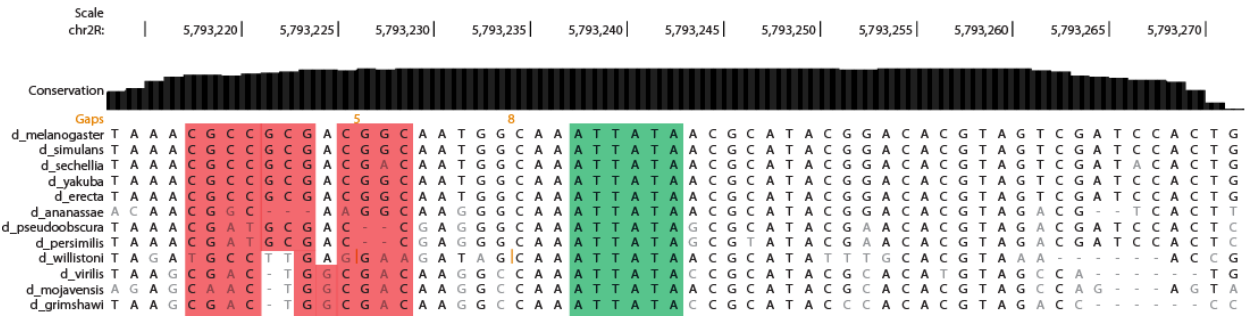

E

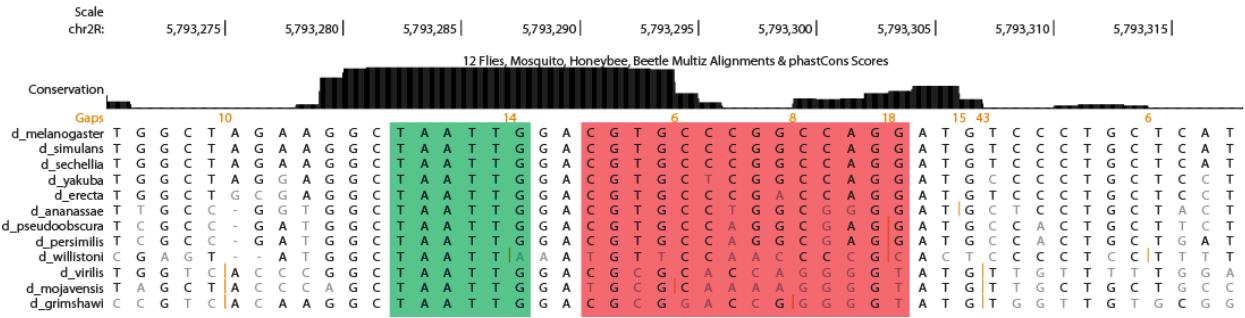

Supplement: S3 Fig — Mad sites are highlighted in red, Medea sites in yellow, Homeodomain sites in green throughout this Fig. The only consensus Mad site (MadD) is shown in purple. (A) Whole Tv4-enhancer showing conserved HD, Mad and Med regions. (B) Sequence identity of HD-A (green), Mad-A (red), Med-A (yellow) across 12 Drosophila species (C) Sequence identity of Mad-D (purple) across 12 Drosophila species. (D) Sequence identity of HD-B (green) and Mad-B (red) across 12 Drosophila species. (E) Sequence identity of HD-C (green) and Mad-C (red) across 12 Drosophila species (PDF) [file pgen.1005754.s003.pdf]

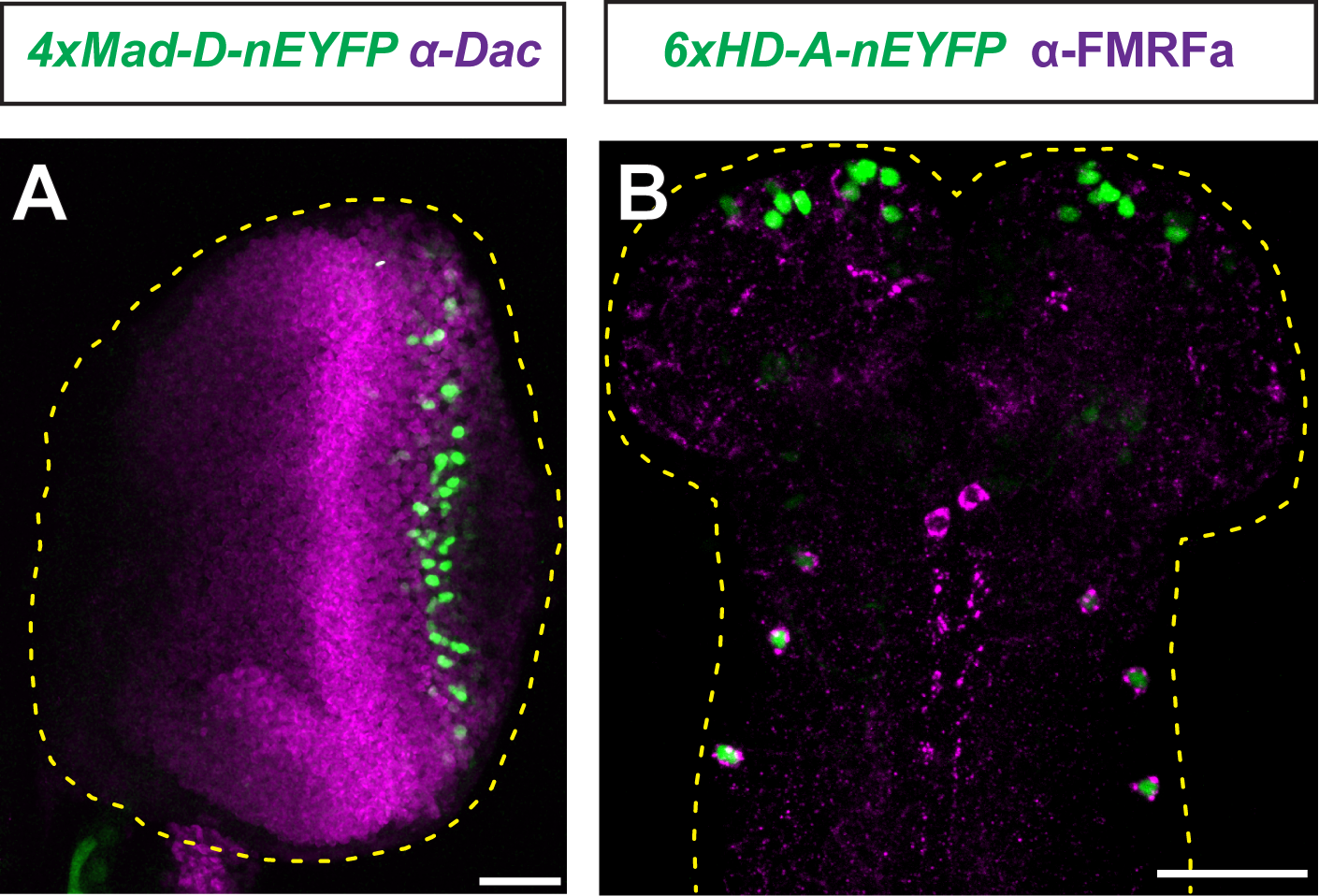

Supplement: S5 Fig — (A) Expression of 4xMad-D-nEYFP in late L3 larvae eye disc. (B) Expression of 6xHD-A-nEYFP in the brain lobes of early L1 larva. Scale bars are 30 μm. (TIF) [file pgen.1005754.s005.tif]

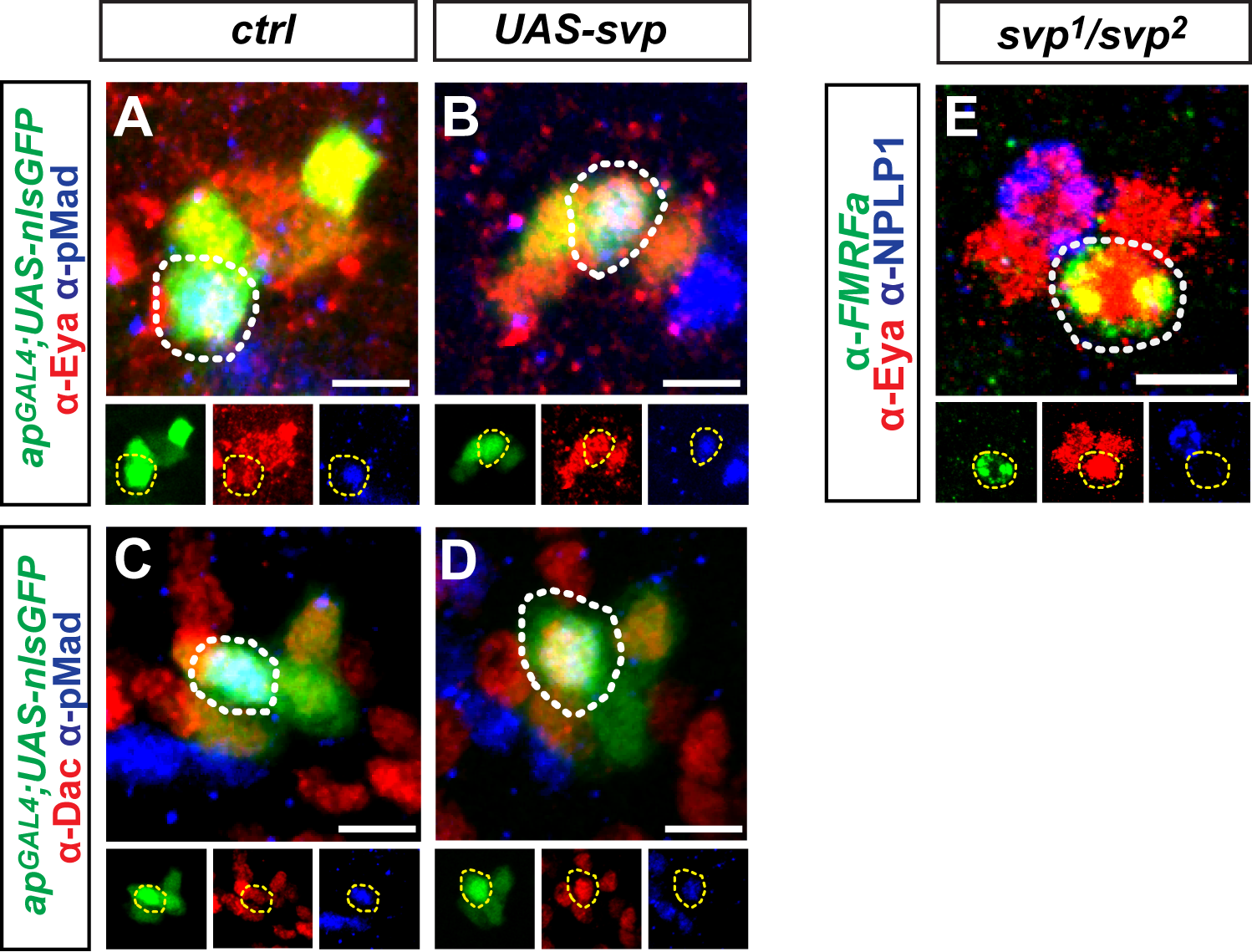

Supplement: S6 Fig — (A-D) Maintaining UAS-svp expression using ap GAL4 does not affect Dac or Eya expression by early L1 larval stages (n = 15 Tv4 neurons per group). (E) Nplp1 and FMRFa expression are unaffected in the Tv clusters of svp 1 /svp 2 animals at late Stg 17. Scale bars represent 5 μm. Dotted circle indicates Tv4 cell. Svp gain of function (ap GAL4 /+;+/+ vs. ap GAL4 /+;+/UAS-svp). (TIF) [file pgen.1005754.s006.tif]

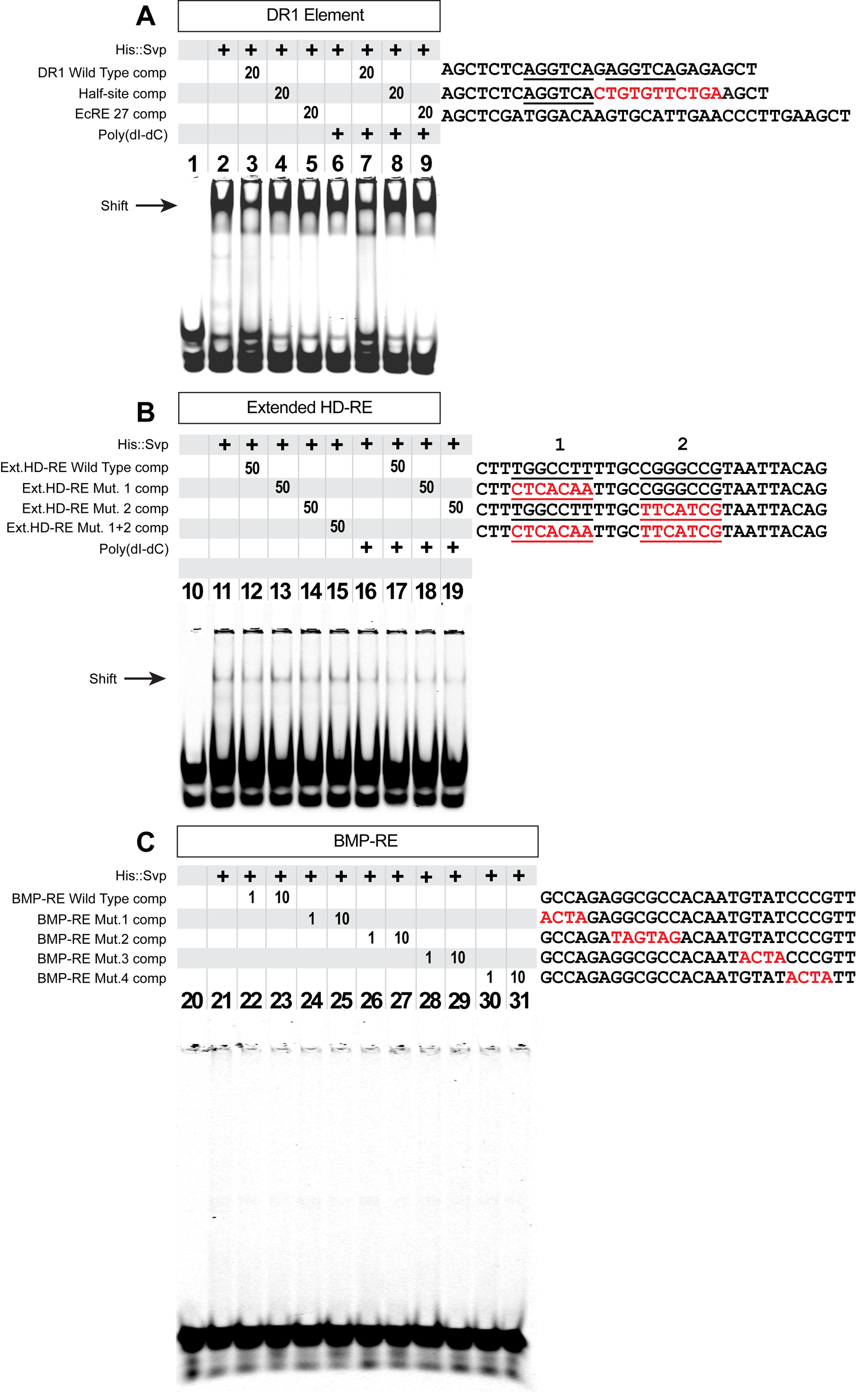

Supplement: S7 Fig — (A) Positive control EMSA using the previously published DR1 Svp binding site oligonucleotides labeled with IRDye700, subjected to binding with purified recombinant His::SVP. Svp binding sites are underlined. The 20:1 stoichiometric ratio of unlabeled competitor to labeled probe is indicated above the lane number in all gels (20). His::Svp generates a strong band shift of the DR1 sequence that is out-competed by wild-type unlabeled competitor (compare Lanes 2,3). Addition of an unlabeled mutated half-Svp site or a non-Svp binding EcR competitor failed to reduce the expected band shift (Lanes 4,5). Addition of poly(dI-dC) does not noticeably affect band shifts (Lanes 6–9). (B) Under the same binding conditions as the DR1 element, addition of His::Svp generated a very weak band shift of the extended HD-RE, containing two putative Svp binding sites that are underlined (Lanes 10,11). Competition by wildtype or mutant unlabeled competitors does not alter His::Svp binding to the labeled probe (Lanes 12–15). Addition of poly(dI-dC) strongly decreases the intensity of the extended HD-RE band shift (Lanes 16–19). (C) Addition of His::Svp does not generate appreciable band shift of the BMP-RE (Lanes 20–31) in any condition. (TIF) [file pgen.1005754.s007.tif]
