# Supplementary material for: Gene Regulatory Mechanisms Underlying the Spatial and Temporal Regulation of Target-Dependent Gene Expression in Drosophila Neurons
Source: PLoS Genet. 2015 Dec 29;11(12):e1005754. doi: 10.1371/journal.pgen.1005754 (PMC4694770; doi:10.1371/journal.pgen.1005754)
Supplement: S4 Fig — Capitalized blue letters denote identity to D.melanogaster Tv4-enhancer sequence. Thick underline denotes BMP-RE. Double underline denotes HD-RE. Red highlight indicates putative Mad-binding sequence. Magenta highlight indicates putative Mad-binding sequence of Mad-D. Green highlight indicates putative Ap-binding sequence. Yellow highlight indicates putative Medea-binding sequence. (PDF) [file pgen.1005754.s004.pdf]

>D. melanogaster

CCATCTGCAGACGTGGTTTTCGAACGTATTTATATTGATTATGGGTGATCGTCA
ACAAGAGCAGTGGACACCCAATAAACCTGTCCAAAAACCCGACACATTTCTGC
CCAGTCATGCGTGGTGGACAATAGCCAAATGCCATTGATGAGACTCGTCTCCA
AAACTTTGGCCTTTTGCCGGGCCGTAATTACAGACTTCCGTCTTTTGAACAGTT
TTTTCAGCCCCACCCAAGAGTCGAGTCTTGAAAAGCTGGCTGGGATGGGGTGG
TTTCGGGTGCTGGACGAGATGCCAGAGGCGCCACAATGTATCCTGTTACAGGT
TACAGGGCCATAAAGCGCCATAAACGCCGCGACGGCAATGGCAAATTATAACG
CATACGGACACGTAGTCGATCCACTGGCTAGAAGGCTAATTGGACGTGCCCGG
CCAGGATGTCCCTGCTCAT

>D. simulans

acgacttggcctggtcatcaaacccaagccaagatttgaacttgaactagtctcgccttatcacattgccttctaattcgttttcattttc
gattttcgcatctacttatgctaatgaagCCATCTGCAGACGTGGTTTTCGAgCGTATTTATATcG
ATTATGGGTGATCGcCAACgAGcGCAGTGGgCACCCAATAAACCTGgCCAAAAAC
CCGACACATTTCTGCCCAGTCATGCGTGGTGGcCAAaAGCCAAATGCCATTGAT
GAGACTCGTCTCCAAAACTTTGGCCTTTTGCCGGGCCGTAATTACAGACTTCCG
TCTTTTGAACAGTTTcTCAGCCCCACCCAAGAGgCGAGTCTTGAAAAGCTGGCgG
GGATGGGGTGGTTTCGGGTGCTGGACGAGATGCCAGAGGCGCCACAAcGTATC
CcGTTACAGGTTACAGGGCCATAAAGCGCCATAAACGCCGCGACGGCAATGGC
AAATTATAACGCATACGGACACGTAGTCGATCCACTGGCTAGAAGGCTAATTG
GACGTGCCCGGCCAGGATGTCCCTGCTCATcccgcagccgacttaaaagtgggcgtaacataacaat
tgtgggcgatttgcatacaatttggtgctcgagtggccagcaaactggcgtcgaaatgttgctacgtcattgttgctgcctct

>D. sechellia

cgcagttggcctggtcatcaaacccaagccaagatttgaacttgacctagtctcgtcttatcacattgccttctaatttgttttcattttc
gattttcgcatctacttatgctaatgaagCCATCTGCAGACGTGGTTTTCGAgCGTATTTATATcG
ATTATGtGTGATCGcCAACgAGcGCAGTGGACACCCAATAAACCTGTCCAAAAAC
CCGACACATTTCTGCCCAGTCATGCGTGGTGGcCAAaAGCCAAATGaCATTGAT
GAGACTCGTCTCCAAAACTTTGGCCTTTTGCCGGGCCGTAATTACAGACTTCCG
TCTTTTGAACAGTTTcTCAGCCCCACCCAAGAGgCGAGTCTTGAAAAGCTGGCgG
GGatATGGGGTGGTTTCGGaTGCTGGACGAGATGCCAGAGGCGCCACAAcGTAT
CCcGTTACAGGTTACAGGGCCATAAAGCGCCATAAACGCCGCGACGaCAATGGC
AAATTATAACGCATACGGACACGTAGTCGATaCACTGGCTAGAAGGCTAATTGG
ACGTGCCCGGCCAGGATGTCCCTGCTCATcccacagccgacttaaaagtgggcgtaacataacaattg
tgggcgatttgcatacaatttggtgctcgagtggccagcaaactggcgtcgaaatgttgctacggcattgttgctgcctct

>D. erecta

**cgacttggcctggtcatcaaaccccaaaccaaaatttgaacttgaactagtctgggcttatcacattgccttctaattcgttttcattttc
gacttttgcatctatttatgctaatgaagCCAaCTGCAtACGTGGTTTcCGcgCGTgTTTATATTGAT
TTGGaTGATCGTaAAGAgcGCAGTGGAtACCCAATAAACCTGgCCAAAACCCGAC
ACATTTCTGCCCAGTCATGCGTGcTGGcCAAaAGCgAAATGCCATTGATGAGACT
CGTCTCCgAAACTTTGGCCTTTTGCCGGGCCGTAATTACAGACTTCCGcCTTTTG
AACAGTTTcTCAGCCCCACCCAAGAGgCGAGTCTTGAAAAGCTGGCgGGGATGGt
gtGTGGTTTgGGGTGCTGGAtGAGATGCCAGAGGCGCCACAAcGTATCCcGTTAC
AGGTTACAGGGCCATAAAGCGaCATAAACGCCGCGACGGCAATGGCAAATTAT
AACGCATACGGACACGTAGTCGATCCACTGGCTgcgAGGCTAATTGGACGTGCC
CGaCCAGGATGTCCCTGCTCcTcccacccgcgacttaaaagtgggcgtaacataacaattgtgggcgatttgca
tacaatttggtgctcgagtggccagcagactggcgtcgaaatgttgctacgtcattgttgccgctttt**

>D. yakuba

**aataaacatcaaatttatttaaatttgaagatacttatatcattgagacatagaaaggaattcaaatgcaaatgaatgaacaataaa
ggcttctggagtgagtccatagattacagagcaACGTGGTTTTCtcgaccagatcataatttgtagctgaactattttcaa
ctggccgattggcacacgacttggcctggtcatcaaacccaaagctaaatttgaacttgaactagtctcgcctcatcacattgccttct
tcgccttcattcgttttcatttccgatttttgcatctatttatgctaataaagccaactgcagacgtggtttctGAgCGagTTTAT
ATTGATTtTtGGTGATCGTAAAGAgcGCAGTGGACACaCAATAAAgCTGgCCAAAA
ACCCGACcCATTTCTGCCCAGTCATGCGTGcTGGcCAAgAGaCAAAGCCATTGAT
GAGACTCGTCTCCAAAACTTTGGCCTTTTGCCGGGCCGTAATTACAGACTTCCG
cCTTTTGAgCAGTTTcTCAGCCCCACCCAAGAGgCGAGTCTTGAAAAGCTGGCggg
gatggggTGGGATGGGGTGGgTTgGGGTGCTGGAtGAGATGCCAGAGGCGCCACAA
cGTATCCcGTTACAGGTTACAGGGCCATAAAGCGCCATAAACGCCGCGACGGCA
ATGGCAAATTATAACGCATACGGACACGTAGTCGATCCACTGGCTAGgAGGCTA
ATTGGACGTGCtCGGCCAGGATGcCCCTGCTCcTcccactggcgacttaaaagtgggcgtagcataa
caattgtgggcgatttgcatacaatttggtactcgagtggccagcaggctggcgtcgaaatgttgctacgtcattgttgctgctttt**

>*D. ananassae*
**ttatgcaaatggagccatcggtctggtggttataaaatttgtatattgatttccgaagatcgtaaagagcgcagctgaacaataatct
tgacggacacgagacgacttggtcttctgaacTTCTGCCCAGTCATGtGTGCCATTGAcggggaaacaact
ggagcttgtcttcgactggtctttgtAACTttgTTGGCCTTtaagTTgGCCCCTAATTACAGgCTTCC
GcaTTTTGAACAGTTTTgcgGCCCCACCCAAGgGgCGAGgCTTaAAAACTGGgcgatT
GGGcactgaacataGGGCTGGACtgggtctggagtggggtggtgggggtgactTGCCAcAGGCGCCAC
AATGTATCCcGTTACAGagctGCGacaACGGCAAggcaagGGCAAATTATAACGCATA
CGGACACGTAGacgtcacttgccggtGGCTAATTGGACGTGCCtGGCggGGATtgcttggctcgt
ccagctcctgctactcctgaagctggcaatcgccttaaaaatgggcgtaccataacaattgtgggcgatttgcatacaatttggctct
caagtggccagaacactggc**

>*D. persimilis*
**gcaaatgaagccatcagcatgtttaagtatccaaactggtttctattgatttcggacgatcgtaaagtgagcatccaacacattaagc
cagacgacaagcagatacatgacaatggacctGACACATTTCTGCCtcaTCATGCccaGTGGACAAA
GtCATTGATaaaacgggcatagtacgtacgtacaagaaacaacacgcacagatacagagacagaattgggctaactggc
AGACTtGTCTtCAAAACTTTGGCCTTTcaaagtgtGGCCcTAATTAtAGACTTCCGCcT
TTGAACAGTTTcgCAGCCCCACCCAAGAGgaggggcctgagacaaagacagggactgggacagaggg
acagagacaggcctgaaaaatgctTGCCAcAGGCGCCACAAcGTATCCcGTTACAtGGCCATAA
AGCtCtATAAACGatGCGACcgagGGCAAATTATAgCGtATACGaACACGTAGaCGAT
CCACTcgccgatGGCTAATTGGACGTGCCaGGCgAGGctactccttcCCCTGCTgATgccactg
ctgatgccactgctgctgccccggctgctgccccggctactgctccggctgctgctgttccttctggaacttaaaagcgggcgtaaca
taacaattgtgggcgatttgcatac**

>*D. pseudoobscura*
**ggacctgacacatttctgcctcatcatgcccagtggacaaagtcattgataaaacgggcatagtacgtacgtacaagaaacaacac
gcacatatacagagacagaagtgggctaactggcAGACTtGTCTtCAAAACTTTGGCCTTTcaaagtgtG
GCCcTAATTAtAGACTTCCGcCTTTGAACAGTTTcgCAGCCCCACCCAAGAGgagggg
cctgagacaaagacagggacagggacagggctgaaaaatgctTGCCAcAGGCGCCACAAcGTATCCcGT
TACAtGGCCATAAAGCtCtATAAACGatGCGACcgagGGCAAATTATAgCGCATACGa
ACACGTAGaCGATCCACTcgccgatGGCTAATTGGACGTGCCaGGCgAGGctactccttcC
CCTGCTgATgccactgcttctgccccggctgctgccccggctgctgccccggctgctgctccggctactgctgttccttctgga
acttaaaagtgggcgtaacataacaattgtgggcgatttgcatac**

>*D. virilis*
**catgcatatttatcgatgcaaatgtctggctattgattttcgacgatcgttagagaacaaaaaaaaaaagaagctaaaatgaaata
agaattgaagcgaactgccagacacacaatggttGACACATTTCTtGCaAGTCATGgtccacatcttggccgga
cgctgggcgggttcttcaaattcagacacgcaaaaaaaataagaaacgggttgaaaacaaAAAACTTGGCCTTTca
aagtgtGGCCGTAATTACAGACTTCCGCTTacgaaaccgaaagtgtccgcccagccaaatggccaagaag
tattgaccgaaGATGGGTtGGgactgggcgcagTCGGGcaGACGctttgggcTGCCAaAGGCGCC
ACAATGTATCCcGTTACAGGGgCCATAAAaCtCCATAAGCGACtGGCgacAAgGcCA
AATTATAcCGCATACGcACAtGTAGccatggtctaagttgtggacccGGCTAATTGGACGcgcacc
aggggtatggtcaggggggtggtggggttgtttttggattctctataaacttaaaagtgggcggcacataacaattgtgggcgatttgc
atacaatttggcgcttggattaccag**

>*D. willistoni*

agtcattgataaaaagcacaagaatgtagaagaagatgaagaagaacaaacacgaataaaaaaaaaactactatctcacacag
tcagtgtcgccaaagttggtctttcaaagtgagggcaTAATTACAGACTTCCGCTTTcaattcgaatgttgtgggc
cACAGTTTagAGAGTCcttttgccagcgGATGCCtgtCGCCACAAgGTATCCatTTACAGtctc
tatagatgccttgagaagaggaagatagaagacgaaCAAATTATAACGCATAtttgCACGTAaaaccgagta
tggctaatttttactaagaaagaaaatgttccaaccccgcagcccacccactcccctccgtactgttttctctcgcccaatttcaaaaa
agtgggcgtaacataacaatt

>*D. mojavensis*

catttgttgccagtcatcctcaccgactgggagtgttgctccgaaatgccaacaacaacaacaacagcaacagaaactaagaaac
aaaaaatcgaaagacgaaaacaaatagaagcgactAAACTTGGCCTTTcaaagtgtGGCCGTAATTACA
GACTTCCGctagctgcaaacgcccgcccagccaaaagagagaggggagggcaaagcgagtagctgagagagaagggg
cagagagagagagagagaggagattgggggattgggttatagcagacgaatgggacaactaaAGGCGCCACAAg
GTATCCcGTTACAGGGCCATAAAacttcaataagagcaactgGCGACAAgGcCAAATTATAA
CGCATACGcACACGTAGccagagtagCTGaaGCTGAAGacccaGCTAATTGGAtgcgataaga
caaaaggggtatggcaggggtagttgataacgctgagggtgttggggtattgctgctgccacagctgacaaaacttaaaagtggg
cggcgcataacaatttgcatacaa

>*D. grimshawi*

cgaacaagcagacaataaatagtgacacatttgtggcaattcaacaatgcctgtctctttgctcagggctgggagagttcttcgaatt
cacacacgccaaagagaaaagacaaagaagagTTTaGCCTTTGaGGCCGTAATTACAGACTTtaact
cactggccaaaaagtgctaaacattcaggccaaaatgcagacactggccaaggaggcggggctgttgctgcgacaggcaacatc
cgacagacggcgcacgccggacgtcaGACGAatcggGATGCtgaAGGCGCCACAATGTATCCcGTT
ACAGGaCCATAAAactctctaaGCGACtGGCgacAAgGcCAAATTATAcCGCATACccACA
CGTAGaccccgtccaagttgtgcacAAGGCTAATTGGACGcggaccgctcttcatggggtatgggttggatatag
gcagatatataaatatataggggatggggaatggttgtgcgggtcttaacttaaaagaaggcggaacataacaattgtgggtgat
