# Supplementary material for: Gene Regulatory Mechanisms Underlying the Spatial and Temporal Regulation of Target-Dependent Gene Expression in Drosophila Neurons
Source: PLoS Genet. 2015 Dec 29;11(12):e1005754. doi: 10.1371/journal.pgen.1005754 (PMC4694770; doi:10.1371/journal.pgen.1005754)
Supplement: S1 Table — (DOCX) [file pgen.1005754.s008.docx]

Table S1. Summary of Tv4 enhancer mutations

| **Descriptor** | **Sequence** |
| --- | --- |
| 445 bp Tv4 FMRFa enhancer | CCATCTGCAGACGTGGTTTTCGAACGTATTTATATTGATTATGGGTGATCGTCAACAAGAGCAGTGGACACCCAATAAACCTGTTCAAAAACCCGACACATTTCTGCCCAGTCATGCGTGGTGGACAATAGCCAAATGCCATTGATGAGACTCGTCTCCCAAACTTTGGCCTTTTGCCGGGCCGTAATTACAGACTTCCGTCTTTTGAACAGTTTTTTCAGCCCCACCCAAGAGTCGAGTCTTGAAAAGCTGGCTGGGATGGGGTGGTTTCGGGTGCTGGACGAGGTGCCAGAGGCGCCACAATGTATCCCGTTACAGGTTACAGGGCCATAAAGCGCCATAAACGCCGCGACGGCAATGGCAAATTATAACGCATACGGACACGTAGTCGATCCACTGGCTAGAAGGCTAATTGGACGTGCCCGGCCAGGATGTCCCTGCTCAT |
| del5' to MadA->End | AAACTTTGGCCTTTTGCCGGGCCGTAATTACAGACTTCCGTCTTTTGAACAGTTTTTTCAGCCCCACCCAAGAGTCGAGTCTTGAAAAGCTGGCTGGGATGGGGTGGTTTCGGGTGCTGGACGAGATGCCAGAGGCGCCACAATGTATCCCGTTACAGGTTACAGGGCCATAAAGCGCCATAAACGCCGCGACGGCAATGGCAAATTATAACGCATACGGACACGTAGTCGATCCACTGGCTAGAAGGCTAATTGGACGTGCCCGGCCAGGATGTCCCTGCTCAT |
| del5' to MadF->End | AACGCCGCGACGGCAATGGCAAATTATAACGCATACGGACACGTAGTCGATCCACTGGCTAGAAGGCTAATTGGACGTGCCCGGCCAGGATGTCCCTGCTCAT |
| del-MadA->MedB island (del-HD-RE) | GCCATCTGCAGACGTGGTTTTCGAACGTATTTATATTGATTATGGGTGATCGTCAACAAGAGCAGTGGACACCCAATAAACCTGTTCAAAAACCCGACACATTTCTGCCCAGTCATGCGTGGTGGACAATAGCCAAATGCCATTGATGAGACTCGTCTCCAAAACAGTTTTTTCAGCCCCACCCAAGAGTCGAGTCTTGAAAAGCTGGCTGGGATGGGGTGGTTTCGGGTGCTGGACGAGATGCCAGAGGCGCCACAATGTATCCCGTTACAGGTTACAGGGCCATAAAGCGCCATAAACGCCGCGACGGCAATGGCAAATTATAACGCATACGGACACGTAGTCGATCCACTGGCTAGAAGGCTAATTGGACGTGCCCGGCCAGGATGTCCCTGCTCAT |
| del-MadD island (del-BMP-RE) | CCATCTGCAGACGTGGTTTTCGAACGTATTTATATTGATTATGGGTGATCGTCAACAAGAGCAGTGGACACCCAATAAACCTGTTCAAAAACCCGACACATTTCTGCCCAGTCATGCGTGGTGGACAATAGCCAAATGCCATTGATGAGACTCGTCTCCAAAACTTTGGCCTTTTGCCGGGCCGTAATTACAGACTTCCGTCTTTTGAACAGTTTTTTCAGCCCCACCCAAGAGTCGAGTCTTGAAAAGCTGGCTGGGATGGGGTGGTTTCGGGTGCTGGTTACAGGGCCATAAAGCGCCATAAACGCCGCGACGGCAATGGCAAATTATAACGCATACGGACACGTAGTCGATCCACTGGCTAGAAGGCTAATTGGACGTGCCCGGCCAGGATGTCCCTGCTCAT |
| Mad-A->Mad-D | AAACTTTGGCCTTTTGCCGGGCCGTAATTACAGACTTCCGTCTTTTGAACAGTTTTTTCAGCCCCACCCAAGAGTCGAGTCTTGAAAAGCTGGCTGGGATGGGGTGGTTTCGGGTGCTGGACGAGATGCCAGAGGCGCCACAATGTATCCCGTTACA |
| 1XHD-RE+1XBMP-RE | TTGCCGGGCCGTAATTACAGACTTCGCTAGAGGACGAGATGCCAGAGGCGCCACAATGTATCC**C**GTTACA |
| Mad-A->Mad-D GC content of spacer is preserved (every other base is compliment; A>T, T>A, C>G, and G>C) | AAACTTTGGCCTTTTGCCGGGCCGTAATTACAGACTTCCGTCTTTTGATCTGATATATGACCGCGAGCGATGTGACCACTGTAGTATACCAGCCAGCGTTCGCGAGCTATGGCGAGGTGGACGAGATGCCAGAGGCGCCACAATGTATCCCGTTACA |
| Mad-A->Mad-D noncomplementary transversion of spacer | AAACTTTGGCCTTTTGCCGGGCCGTAATTACAGACTTCCGTCTTTTGCCACTGGGGGGACTAAAACAAACCTCTGATCTGAGGTCCCCTAGTTAGTTTCGTTTTGTTGGGATTTGTAGGGACGAGATGCCAGAGGCGCCACAATGTATCCCGTTACA |
| ΔMadA | CCATCTGCAGACGTGGTTTTCGAACGTATTTATATTGATTATGGGTGATCGTCAACAAGAGCAGTGGACACCCAATAAACCTGTTCAAAAACCCGACACATTTCTGCCCAGTCATGCGTGGTGGACAATAGCCAAATGCCATTGATGAGACTCGTCTCCCAAACTTTGGCCTTTTAGTAGTACGTAATTACAGACTTCCGTCTTTTGAACAGTTTTTTCAGCCCCACCCAAGAGTCGAGTCTTGAAAAGCTGGCTGGGATGGGGTGGTTTCGGGTGCTGGACGAGGTGCCAGAGGCGCCACAATGTATCCCGTTACAGGTTACAGGGCCATAAAGCGCCATAAACGCCGCGACGGCAATGGCAAATTATAACGCATACGGACACGTAGTCGATCCACTGGCTAGAAGGCTAATTGGACGTGCCCGGCCAGGATGTCCCTGCTCAT |

| **Descriptor** | **Sequence** |
| --- | --- |
| ΔHD-A | CCATCTGCAGACGTGGTTTTCGAACGTATTTATATTGATTATGGGTGATCGTCAACAAGAGCAGTGGACACCCAATAAACCTGTTCAAAAACCCGACACATTTCTGCCCAGTCATGCGTGGTGGACAATAGCCAAATGCCATTGATGAGACTCGTCTCCCAAACTTTGGCCTTTTGCCGGGCCGGAGCTCCAGACTTCCGTCTTTTGAACAGTTTTTTCAGCCCCACCCAAGAGTCGAGTCTTGAAAAGCTGGCTGGGATGGGGTGGTTTCGGGTGCTGGACGAGGTGCCAGAGGCGCCACAATGTATCCCGTTACAGGTTACAGGGCCATAAAGCGCCATAAACGCCGCGACGGCAATGGCAAATTATAACGCATACGGACACGTAGTCGATCCACTGGCTAGAAGGCTAATTGGACGTGCCCGGCCAGGATGTCCCTGCTCAT |
| ΔMedA+B | CCATCTGCAGACGTGGTTTTCGAACGTATTTATATTGATTATGGGTGATCGTCAACAAGAGCAGTGGACACCCAATAAACCTGTTCAAAAACCCGACACATTTCTGCCCAGTCATGCGTGGTGGACAATAGCCAAATGCCATTGATGAGACTCGTCTCCCAAACTTTGGCCTTTTGCCGGGCCGTAATTACGAACTTCCGTTCTTTGAACAGTTTTTTCAGCCCCACCCAAGAGTCGAGTCTTGAAAAGCTGGCTGGGATGGGGTGGTTTCGGGTGCTGGACGAGGTGCCAGAGGCGCCACAATGTATCCCGTTACAGGTTACAGGGCCATAAAGCGCCATAAACGCCGCGACGGCAATGGCAAATTATAACGCATACGGACACGTAGTCGATCCACTGGCTAGAAGGCTAATTGGACGTGCCCGGCCAGGATGTCCCTGCTCAT |
| ΔMadD | CCATCTGCAGACGTGGTTTTCGAACGTATTTATATTGATTATGGGTGATCGTCAACAAGAGCAGTGGACACCCAATAAACCTGTTCAAAAACCCGACACATTTCTGCCCAGTCATGCGTGGTGGACAATAGCCAAATGCCATTGATGAGACTCGTCTCCCAAACTTTGGCCTTTTGCCGGGCCGTAATTACAGACTTCCGTCTTTTGAACAGTTTTTTCAGCCCCACCCAAGAGTCGAGTCTTGAAAAGCTGGCTGGGATGGGGTGGTTTCGGGTGCTGGACGAGGTGCCAGATAGTAGACAATGTATCCCGTTACAGGTTACAGGGCCATAAAGCGCCATAAACGCCGCGACGGCAATGGCAAATTATAACGCATACGGACACGTAGTCGATCCACTGGCTAGAAGGCTAATTGGACGTGCCCGGCCAGGATGTCCCTGCTCAT |
| ΔMadE+F | CCATCTGCAGACGTGGTTTTCGAACGTATTTATATTGATTATGGGTGATCGTCAACAAGAGCAGTGGACACCCAATAAACCTGTTCAAAAACCCGACACATTTCTGCCCAGTCATGCGTGGTGGACAATAGCCAAATGCCATTGATGAGACTCGTCTCCCAAACTTTGGCCTTTTGCCGGGCCGTAATTACAGACTTCCGTCTTTTGAACAGTTTTTTCAGCCCCACCCAAGAGTCGAGTCTTGAAAAGCTGGCTGGGATGGGGTGGTTTCGGGTGCTGGACGAGGTGCCAGAGGCGCCACAATGTATCCCGTTACAGGTTACAGGGCCATAGTAGTAGTAGTAGTAGTCGACGGCAATGGCAAATTATAACGCATACGGACACGTAGTCGATCCACTGGCTAGAAGGCTAATTGGACGTGCCCGGCCAGGATGTCCCTGCTCAT |
| ΔMadB | CCATCTGCAGACGTGGTTTTCGAACGTATTTATATTGATTATGGGTGATCGTCAACAAGAGCAGTGGACACCCAATAAACCTGTTCAAAAACCCGACACATTTCTGCCCAGTCATGCGTGGTGGACAATAGCCAAATGCCATTGATGAGACTCGTCTCCCAAACTTTGGCCTTTTGCCGGGCCGTAATTACAGACTTCCGTCTTTTGAACAGTTTTTTCAGCCCCACCCAAGAGTCGAGTCTTGAAAAGCTGGCTGGGATGGGGTGGTTTCGGGTGCTGGACGAGGTGCCAGAGGCGCCACAATGTATCCCGTTACAGGTTACAGGGCCATAAAGCGCCATAAAGTAGTAGTAGTAAATGGCAAATTATAACGCATACGGACACGTAGTCGATCCACTGGCTAGAAGGCTAATTGGACGTGCCCGGCCAGGATGTCCCTGCTCAT |
| ΔHD-B | CCATCTGCAGACGTGGTTTTCGAACGTATTTATATTGATTATGGGTGATCGTCAACAAGAGCAGTGGACACCCAATAAACCTGTTCAAAAACCCGACACATTTCTGCCCAGTCATGCGTGGTGGACAATAGCCAAATGCCATTGATGAGACTCGTCTCCCAAACTTTGGCCTTTTGCCGGGCCGTAATTACAGACTTCCGTCTTTTGAACAGTTTTTTCAGCCCCACCCAAGAGTCGAGTCTTGAAAAGCTGGCTGGGATGGGGTGGTTTCGGGTGCTGGACGAGGTGCCAGAGGCGCCACAATGTATCCCGTTACAGGTTACAGGGCCATAAAGCGCCATAAACGCCGCGACGGCAATGGCAAGAGCTCACGCATACGGACACGTAGTCGATCCACTGGCTAGAAGGCTAATTGGACGTGCCCGGCCAGGATGTCCCTGCTCAT |
| ΔHDC | CCATCTGCAGACGTGGTTTTCGAACGTATTTATATTGATTATGGGTGATCGTCAACAAGAGCAGTGGACACCCAATAAACCTGTTCAAAAACCCGACACATTTCTGCCCAGTCATGCGTGGTGGACAATAGCCAAATGCCATTGATGAGACTCGTCTCCCAAACTTTGGCCTTTTGCCGGGCCGTAATTACAGACTTCCGTCTTTTGAACAGTTTTTTCAGCCCCACCCAAGAGTCGAGTCTTGAAAAGCTGGCTGGGATGGGGTGGTTTCGGGTGCTGGACGAGGTGCCAGAGGCGCCACAATGTATCCCGTTACAGGTTACAGGGCCATAAAGCGCCATAAACGCCGCGACGGCAATGGCAAATTATAACGCATACGGACACGTAGTCGATCCACTGGCTAGAAGGCGAGCTCGACGTGCCCGGCCAGGATGTCCCTGCTCAT |
| ΔMadC | CCATCTGCAGACGTGGTTTTCGAACGTATTTATATTGATTATGGGTGATCGTCAACAAGAGCAGTGGACACCCAATAAACCTGTTCAAAAACCCGACACATTTCTGCCCAGTCATGCGTGGTGGACAATAGCCAAATGCCATTGATGAGACTCGTCTCCCAAACTTTGGCCTTTTGCCGGGCCGTAATTACAGACTTCCGTCTTTTGAACAGTTTTTTCAGCCCCACCCAAGAGTCGAGTCTTGAAAAGCTGGCTGGGATGGGGTGGTTTCGGGTGCTGGACGAGGTGCCAGAGGCGCCACAATGTATCCCGTTACAGGTTACAGGGCCATAAAGCGCCATAAACGCCGCGACGGCAATGGCAAATTATAACGCATACGGACACGTAGTCGATCCACTGGCTAGAAGGCTAATTGGAAGTAGTAGTAGTAGATGTCCCTGCTCAT |
| 6xHD-RE | TTGCCGGGCCGTAATTACAGACTTCGCTAGATTGCCGGGCCGTAATTACAGACTTCGCTAGATTGCCGGGCCGTAATTACAGACTTCGCTAGATTGCCGGGCCGTAATTACAGACTTCGCTAGATTGCCGGGCCGTAATTACAGACTTCGCTAGATTGCCGGGCCGTAATTACAGACTTCG |

| **Descriptor** | **Sequence** |
| --- | --- |
| 4XSpacer element | AACAGTTTTTTCAGCCCCACCCAAGAGTCGAGTCTTGAAAAGCTGGCTGGGATGGGGTGGTTTCGGGTGCTGCTAGAAACAGTTTTTTCAGCCCCACCCAAGAGTCGAGTCTTGAAAAGCTGGCTGGGATGGGGTGGTTTCGGGTGCTGAATTCAACAGTTTTTTCAGCCCCACCCAAGAGTCGAGTCTTGAAAAGCTGGCTGGGATGGGGTGGTTTCGGGTGCTGCTAGAAACAGTTTTTTCAGCCCCACCCAAGAGTCGAGTCTTGAAAAGCTGGCTGGGATGGGGTGGTTTCGGGTGCT |
| 4xBMP-RE | GGACGAGATGCCAGAGGCGCCACAATGTATCCCGTTACAGCTAGAGGACGAGATGCCAGAGGCGCCACAATGTATCCCGTTACAGCTAGAGGACGAGATGCCAGAGGCGCCACAATGTATCCCGTTACAGCTAGAGGACGAGATGCCAGAGGCGCCACAATGTATCCCGTTACA |
| 4XMad-E+F | GGTTACAGGGCCATAAAGCGCCATAAACGCCGCGACGGCAATGCTAGAGGTTACAGGGCCATAAAGCGCCATAAACGCCGCGACGGCAATGCTAGAGGTTACAGGGCCATAAAGCGCCATAAACGCCGCGACGGCAATGCTAGAGGTTACAGGGCCATAAAGCGCCATAAACGCCGCGACGGCAAT |
| 4XMadF->End | AACGCCGCGACGGCAATGGCAAATTATAACGCATACGGACACGTAGTCGATCCACTGGCTAGAAGGCTAATTGGACGTGCCCGGCCAGGATGGCTAGAAACGCCGCGACGGCAATGGCAAATTATAACGCATACGGACACGTAGTCGATCCACTGGCTAGAAGGCTAATTGGACGTGCCCGGCCAGGATGGCTAGAAACGCCGCGACGGCAATGGCAAATTATAACGCATACGGACACGTAGTCGATCCACTGGCTAGAAGGCTAATTGGACGTGCCCGGCCAGGATGGCTAGAAACGCCGCGACGGCAATGGCAAATTATAACGCATACGGACACGTAGTCGATCCACTGGCTAGAAGGCTAATTGGACGTGCCCGGCCAGGATG |

**Mutations in Red**
**Restriction enzyme sites or Restriction scar sites in concatemers in Blue**
